# MARK4 regulates NLRP3 positioning and inflammasome activation through a microtubule-dependent mechanism

**DOI:** 10.1038/ncomms15986

**Published:** 2017-06-28

**Authors:** Xuan Li, Sarah Thome, Xiaodan Ma, Mamta Amrute-Nayak, Alison Finigan, Lauren Kitt, Leanne Masters, John R. James, Yuguang Shi, Guoyu Meng, Ziad Mallat

**Affiliations:** 1 Department of Medicine, University of Cambridge, The West Forvie Building, Robinson Way, Cambridge, CB2 0SZ, UK; 2 State Key Laboratory of Medical Genomics, Shanghai Institute of Hematology, Rui-Jin Hospital affiliated to Shanghai JiaoTong University School of Medicine, 197 Ruijin Er Road, Shanghai 200025, China; 3 Department of Molecular and Cell Physiology, Hannover Medical School, D-30625 Hannover, Germany; 4 Molecular Immunity Unit, Department of Medicine, University of Cambridge, MRC-LMB, Francis Crick Avenue, Cambridge, CB2 0QH, UK; 5 Barshop Institute for longevity and Aging Studies, University of Texas Health Science Center at San Antonio. Texas Research Park Campus MC 7755, 15355 Lamda Drive, San Antonio, Texas 78245, USA; 6 Institut National de la Santé et de la Recherche Médicale, U970, Paris, France

## Abstract

Excessive activation of the NLR family pyrin domain containing 3 (NLRP3) inflammasome is involved in many chronic inflammatory diseases, including cardiovascular and Alzheimer’s disease. Here we show that microtubule-affinity regulating kinase 4 (MARK4) binds to NLRP3 and drives it to the microtubule-organizing centre, enabling the formation of one large inflammasome speck complex within a single cell. MARK4 knockdown or knockout, or disruption of MARK4-NLRP3 interaction, impairs NLRP3 spatial arrangement and limits inflammasome activation. Our results demonstrate how an evolutionarily conserved protein involved in the regulation of microtubule dynamics orchestrates NLRP3 inflammasome activation by controlling its transport to optimal activation sites, and identify a targetable function for MARK4 in the control of innate immunity.

## Abstract (web summary)

Microtubules regulate activation of the NLRP3 inflammasome, but the underlying molecular mechanism is unclear. Here the authors show that MARK4 binds NLRP3 and shuttles it to the microtubule-organizing centre to promote optimal inflammasome activation.

Inflammasomes are important signalling platforms of the innate immune system, poised to detect a wide range of molecular signatures including pathogens and sterile agents. They are multiprotein complexes with a key component consisting of a NOD-like receptor (NLR) family member or Pyrin and Hin domain-containing protein (PYHIN) family member, connected to caspase-1 mostly through an adaptor protein named apoptosis-associated Speck-like protein with a caspase-recruitment domain (ASC, encoded by *Pycard*). Activation of inflammasome pathways triggers caspase-1-dependent processing of immature interleukin 1β (pro-IL-1β) into bioactive IL-1β. Activation of NLRP3, an NLR member of the inflammasome family, is linked to the pathogenesis of various disorders^1^,^2^,^3^. A better understanding of the regulation of NLRP3 inflammasome activation is therefore required for the development of effective therapies to combat a range of inflammatory diseases.

Microtubule-dependent sorting is critical for the subcellular localization of many organelles, vesicles, macromolecules and pathogens^4^. Studies indicate that microtubules regulate intracellular sorting of NLRP3 (refs 5, 6), microtubules mediate assembly of the NLRP3 inflammasome^6^, and microtubule disruption attenuates NLRP3 inflammasome activation^5^,^6^. Data indicate that the centrosomal protein NEK7 is important in the regulation of NLRP3 activity^7^,^8^,^9^, but inhibition of tubulin polymerization does not affect the NLRP3-NEK7 interaction^7^. Thus, the molecular mechanisms through which NLRP3 is sorted in a microtubule-dependent manner, and the functional consequences of these processes, are not well understood.

The microtubule-affinity regulating kinase 4 (MARK4) protein was discovered by its ability to phosphorylate microtubule-associated proteins (MAP) and thereby regulates microtubule dynamics^10^. The cellular functions of MARK4 have mostly been investigated in neurons, but MARK4 has versatile functions that extend beyond the regulation of microtubule dynamics to a variety of cellular processes including cell polarity, organelle positioning, intracellular transport, signal transduction, ciliogenesis and adipogenesis^11^,^12^,^13^,^14^,^15^,^16^.

In this study, we identify a functional requirement of MARK4 in the sorting and activation of the NLRP3 inflammasome. Our results show that delivery of NLRP3 through a MARK4-regulated machinery to the correct position on microtubule-organizing center (MTOC) is also very important in determining NLRP3 activation. We demonstrate that amongst the various NLR members of inflammasome family, MARK4 selectively interacts with NLRP3 and promotes its recruitment to mitochondria. Remarkably, we show that MARK4 drives NLRP3 to MTOC, thereby allowing NLRP3 positioning to the characteristic speck structure in a microtubule-dependent manner, and providing an explanation for the presence of only one inflammasome complex per cell. Furthermore, loss of MARK4 or disruption of the interaction between NLRP3 and MARK4, alters NLRP3 inflammasome activation. Finally, we show that loss of MARK4 reduces neutrophil recruitment upon monosodium urate (MSU)-induced inflammasome activation *in vivo*. Collectively, these data highlight a requirement of MARK4 in facilitating microtubule-based transport of NLRP3, and identify a functional interaction that regulates NLRP3 positioning and optimal activation output. The identification of MARK4 as a NLRP3 binding partner advances our understanding of the molecular mechanisms contributing to inflammasome sorting by microtubule-based transport, and identifies a potential therapeutic target.

## Results

### Loss of MARK4 alters NLRP3-dependent IL-1β production

**Figure 1:**
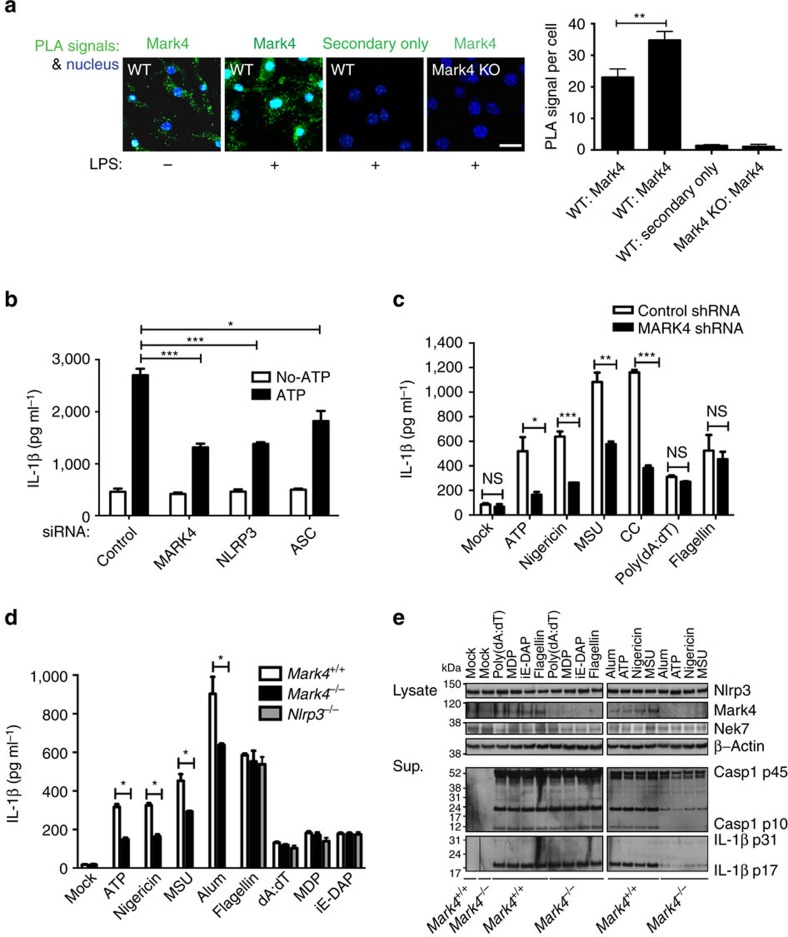
Loss of MARK4 affects IL-1β production under NLRP3 inflammasome activation. (**a**) Mark4 expression under unprimed and LPS primed conditions in mouse bone marrow-derived macrophages (BMDM). Cytoplasm proximity ligation assay (PLA) signal per cell cytoplasm is used for quantification. Mean±s.e.m. for all the cells taken from 5 to 8 different views at × 40 magnification for each group. Experiments have been repeated three times. Scale bar, 10 μm. (**b**) siRNA of MARK4, NLRP3 or ASC caused reduction of ATP induced IL-1β production in THP-1 cells. (**c**) shRNA of MARK4 caused reduction of IL-1β induced by NLRP3 stimuli, including monosodium urate (MSU) and cholesterol crystals (CC) in THP-1 cells. Mock represents macrophages without further stimulation. (**d**) BMDM derived from Mark4 KO mice exhibited selectivity towards NLRP3 activating stimuli. Mean±s.e.m., three to four independent experiments combined (**b**–**d**), comparisons of the two different groups were analysed by unpaired *t* test. NS was considered as not statistically significant. *P<0.05, **P<0.01 and ***P<0.001 were considered as statistically significant (**a**–**d**). (**e**) Representative western blots of caspase-1 (Casp1) and IL-1β in the supernatant (Sup.) in *Mark4*^+/+^ and *Mark4*^−/−^ macrophages activated with indicated stimuli. Cell lysates were used as controls to indicate equal amount of cells for analysis.

Microtubule-depolymerizing agent colchicine is an effective medicine to treat the acute phase of gout inflammatory disease. Recent studies indicate that microtubule-driven spatial arrangement of mitochondria promotes activation of NLRP3 inflammasome^6^. Moreover, colchicine blocks IL-1β production in response to MSU^5^. To confirm the requirement of microtubules in regulating IL-1β production, we showed that in C57BL/6 mouse bone marrow derived macrophages (BMDM), microtubule-disrupting drugs including colchicine, nocodazole, and vinblastine suppressed IL-1β production in response to the NLRP3 inflammasome stimulus ATP (Supplementary Fig. 1). We therefore addressed in detail how microtubule-related functions are linked to the regulation of NLRP3 inflammasome activation. MARK4 is upregulated during permanent focal cerebral ischemia^17^ and in brain tissue during Alzheimer’s disease^18^, indicating a potential regulatory role in the context of sterile inflammation. *Mark4* null mice are protected from diet-induced obesity and insulin resistance^13^, a very similar phenotype to *Nlrp3* null mice^19^. *In situ* proximity ligation assay (PLA) is suitable for quantitative studies of protein expression, protein modifications and close protein interactions^20^,^21^. By using this assay, we found that MARK4 was endogenously expressed in macrophages, including mouse BMDM (Fig. 1a and Supplementary Fig. 2c) and differentiated human THP-1 cells (Supplementary Fig. 2a,b). The expression level of Mark4 was inducible by LPS priming (Fig. 1a and Supplementary Fig. 2c). Under a wide set of NLRP3 stimuli, including ATP, nigericin, or sterile crystals, knock down of MARK4 using small interfering RNAs (siRNA) or short hairpin RNA (shRNA; Supplementary Fig. 2d) significantly reduced IL-1β production (Fig. 1b,c). We further delineated the specificity of Mark4 by demonstrating that *Mark4* knock-out (indicated as Mark4 KO or *Mark4*^−/−^) BMDMs had reduced level of IL-1β production specifically after activation by NLRP3 stimuli when compared with wild-type cells (indicated as WT or *Mark4*^+/+^), but not after incubation with stimuli (for example, flagellin, dA:dT, muramyl dipeptide or iE-DAP) known to induce other inflammasome pathways (Fig. 1d). Upon NLRP3 activation, IL-1β production by Mark4 KO cells was not totally ablated as compared with Nlrp3 KO cells, indicating a regulatory role of MARK4 in optimizing NLRP3 activation, or a partial compensatory/redundant mechanism occurring in *Mark4*^−/−^ BMDMs. Importantly, IL-1β reduction in *Mark4*^−/−^ cells was not caused by the following: altered levels of Nlrp3 (Fig. 1e; Supplementary Fig. 3a,b), pro-caspase-1 or pro-IL-1β substrates (Supplementary Fig. 3); perturbed macrophage differentiation (Supplementary Fig. 4); compromised phagocytosis of crystals (Supplementary Fig. 5); or reduced macrophage viability (Supplementary Fig. 6d,e). Reduced IL-1β levels in *Mark4*^−/−^ BMDM cells after activation of NLRP3 were due to reduced generation of active caspase-1 and active IL-1β (Fig. 1e). Surprisingly, we did not see any difference in cell death rate between WT and Mark4 KO cells upon NLRP3 inflammasome activation (Supplementary Fig. 6d,e). Inhibition of inflammasome-dependent caspase-1 activation and IL-1β is not always associated with reduction of cell death^22^. In the case of Mark4 deficiency, this could be due to residual caspase-1 cleavage, altered cell death kinetics, or existence of alternative cell death control in Mark4 KO cells. In addition, inflammasome pathway-independent TNF production was comparable between wild-type and knock-out cells under different treatment conditions (Supplementary Fig. 6a). These results indicate a specific role of Mark4 in Nlrp3 inflammasome activation.

### MARK4 is primarily associated with NLRP3 inflammasome

**Figure 2:**
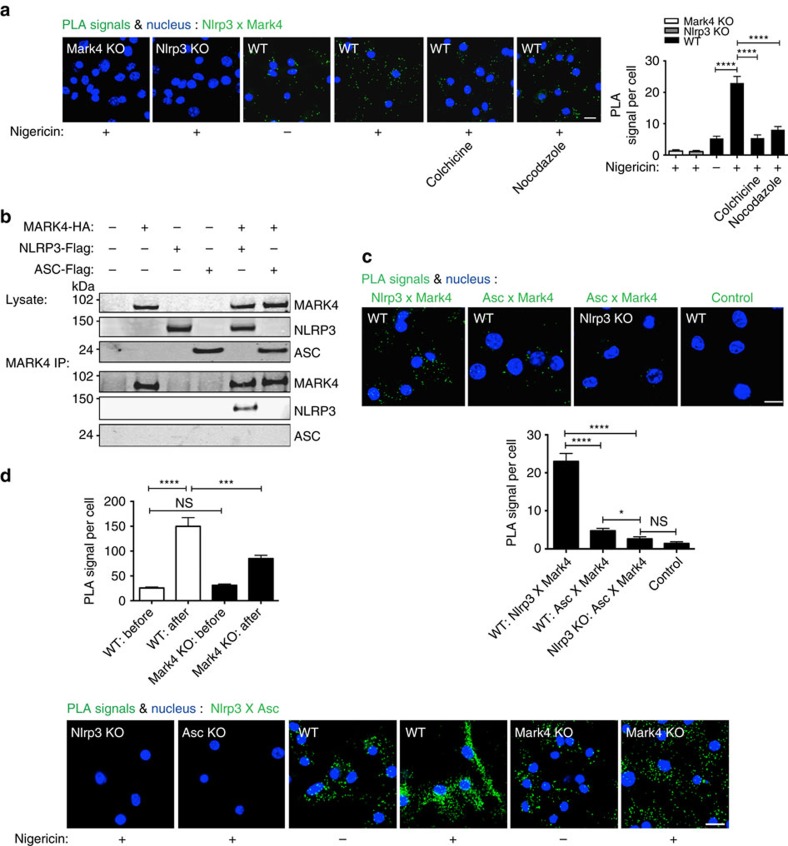
MARK4 interacts with NLRP3 in a microtubule-dependent manner. (**a**) Upon nigericin stimulation (3 μM for 2 h), microtubule-disrupting drugs colchicine and nocodazole reduced the interaction between Mark4 and Nlrp3, shown by PLA signals in WT BMDM cells. Mark4 KO and Nlrp3 KO BMDM cells were employed as controls. (**b**) MARK4 was associated with NLRP3 in co-immunoprecipitation assay. Whole cell lysates were analysed as indication of transfection. Western blots are representative of 3 independent experiments. (**c**) Upon nigericin stimulation, PLA signal of NlrpP3 and Mark4, or Asc and Mark4 in BMDM cells derived from WT or Nlrp3 KO. Secondary only was employed as control in this PLA assay. (**d**) PLA signal of Nlrp3 and Asc in BMDM cells derived from WT or Nlrp3 KO or Asc KO before or after nigericin stimulation (3 μM for 2 h). Mean±s.e.m. for all the cells taken from 5 to 8 different views at × 40 magnification for each group (**a**,**b**,**d**). Comparisons of the two different groups were analysed by unpaired *t*-test. NS was considered as not statistically significant. *P<0.05, ***P<0.001 and ****P<0.0001 were considered as statistically significant (**a**,**c**,**d**). Results are representatives of three independent experiments. Scale bar, 10 μm.

NLRP3 inflammasome complex include NLRP3 itself, ASC adaptor, and caspase-1 (ref. 23). By using PLA to detect protein-protein interaction *in situ*, we found that Mark4 (or MARK4) was associated with Nlrp3 (or NLRP3) in BMDM (Fig. 2a) and THP-1 cells (Supplementary Fig. 7a). This association was enhanced by NLRP3 inflammasome activation (Fig. 2a; Supplementary Fig. 7a). We further found that microtubule-disrupting agents including colchicine and nocodazole significantly reduced the degree of association between Mark4 and Nlrp3 in BMDM cells (Fig. 2a), indicating a requirement for microtubule integrity in mediating this interaction. To further delineate the direct association between MARK4 and components in the inflammasome complex, we co-overexpressed HA-tagged MARK4 respectively with flagged-tagged NLRP3 or ASC in HEK293T cells that lack endogenous inflammasome proteins^24^. Clearly, we found that MARK4 co-immunoprecipitated with NLRP3, but not ASC (Fig. 2b). We also found a greater level of Nlrp3/MarK4 association compared with Asc/Mark4 association in mouse macrophages under the same experimental conditions (Fig. 2c; Supplementary Fig. 8), and Nlrp3 deficiency further reduced Mark4/Asc association to background level (Fig. 2c). As expected, NLRP3 inflammasome activation can instigate enhanced level of Nlrp3/Asc interaction in wild-type cells (Fig. 2d). However, this Nlrp3/Asc association was significantly reduced in Mark4 deficient cells compared with wild-type cells after Nlrp3 inflammasome activation (Fig. 2d), strongly indicating Mark4 was involved in forming NLRP3 complex.

We were also not able to detect any association between MARK4 and other inflammasomes (NLRP1, NLRC4, or Aim2; Supplementary Fig. 7b), which is in line with the lack of any impact of Mark4 deficiency on IL-1β production in response to stimuli targeting those inflammasome pathways (Fig. 1d). Furthermore, mammalian MARK family has 4 members, and NLRP3 was specifically and highly-associated with MARK4 but not with other members of the MARK family (Supplementary Fig. 7c), supporting a specific role of MARK4 in this pathway. Collectively, those data demonstrate a genuine, primary and specific interaction between MARK4 and NLRP3.

**Figure 3:**
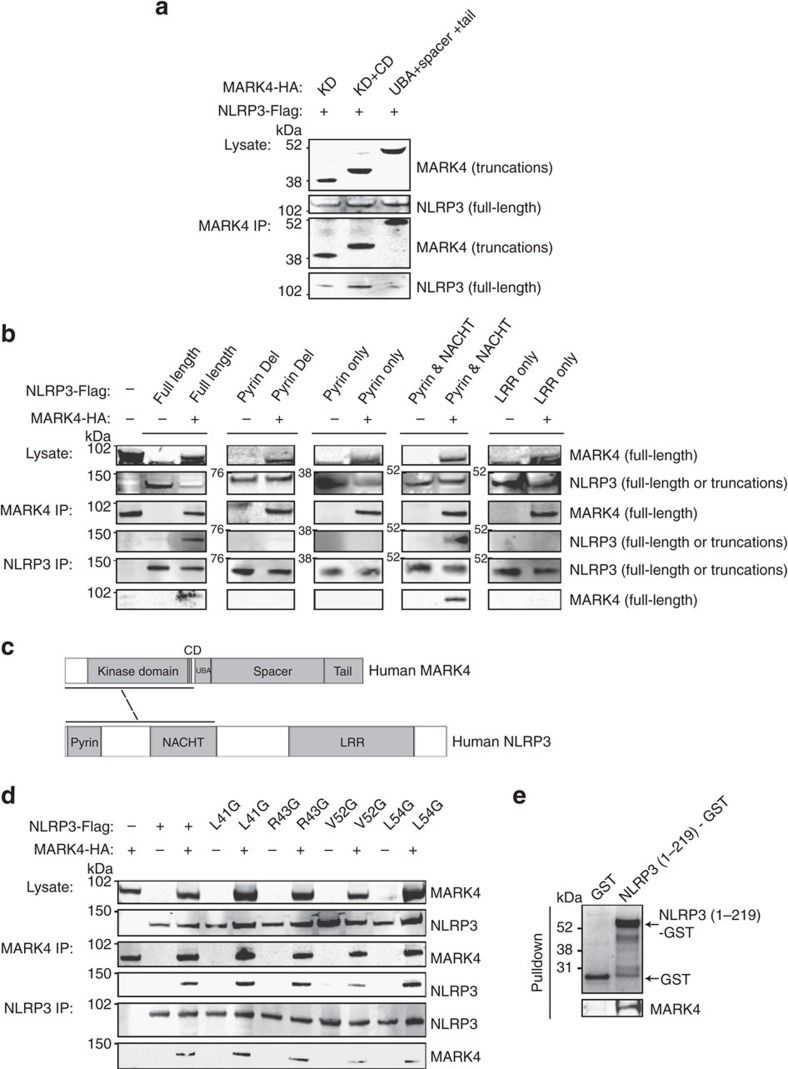
Mapping of interacting domains between MARK4 and NLRP3. (**a**) Western blots of co-immunoprecipitated full-length NLRP3 with truncated MARK4 (KD: kinase domain; CD: catalytic domain; UBA: Ubiquitin-associated domain), respectively. (**b**) Western blots of coimmunoprecipitated truncated NLRP3 (Pyrin Del: pyrin domain deletion; Pyrin only: pyrin domain only; NACHT: Nucleotide-binding oligomerization domain; LRR: Leucine-rich repeat) with full-length MARK4, or co-immunopreciptated MARK4 with truncated NLRP3, respectively. (**c**) Schematic diagram showing that MARK4 catalytic kinase domain and NLRP3 Pyrin &NACHT domain were essential for their interaction. (**d**) Western blots of co-immunoprecipitated NLRP3 point mutants with MARK4 respectively. Whole cell lysates were analysed as indication of transfection. (**e**) Purified recombinant NLRP3 (1-291aa) immobilized on the glutathione sepharose can pull down purified recombinant full-length MARK4 directly. Western blots are representatives of three independent experiments.

MARK4 has a conserved structure consisting of four distinct domains, including a kinase and a catalytic domain^25^,^26^. NLRP3 is composed of a C-terminal leucine-rich repeat domain for sensing the ligands, a central nucleotide-binding domain for oligomerization, and an N-terminal effector pyrin domain (PYD)^23^. The PYD domain of NLRP3 is important for promoting ASC assembly by binding to GBP5 (ref. 22), and controls positioning of NLRP3 to mitochondria by binding to MAVS^27^. To understand the molecular mechanism of NLRP3/MARK4 association, we mapped the interaction between NLRP3 and MARK4 using various truncations of these two proteins. We found that MARK4 catalytic kinase domain and NLRP3 Pyrin-NACHT domains were essential for this interaction (Fig. 3a–c). We therefore mutated to Glycine (G) amino acids of this PYD region (L41, R43, V52, and L54) predicted to bind to MARK4. Interestingly V52G displayed a substantial reduction of binding affinity to MARK4 (Fig. 3d), suggesting an essential role of this region of NLRP3 in binding to MARK4. Furthermore, we demonstrated that purified recombinant MARK4 protein was able to bind to immobilized recombinant NLRP3 protein (1–219 amino acids (aa)) using GST pull-down assay (Fig. 3e), indicating a direct binding between these two proteins.

### MARK4 is involved in NLRP3 positioning along microtubules

**Figure 4:**
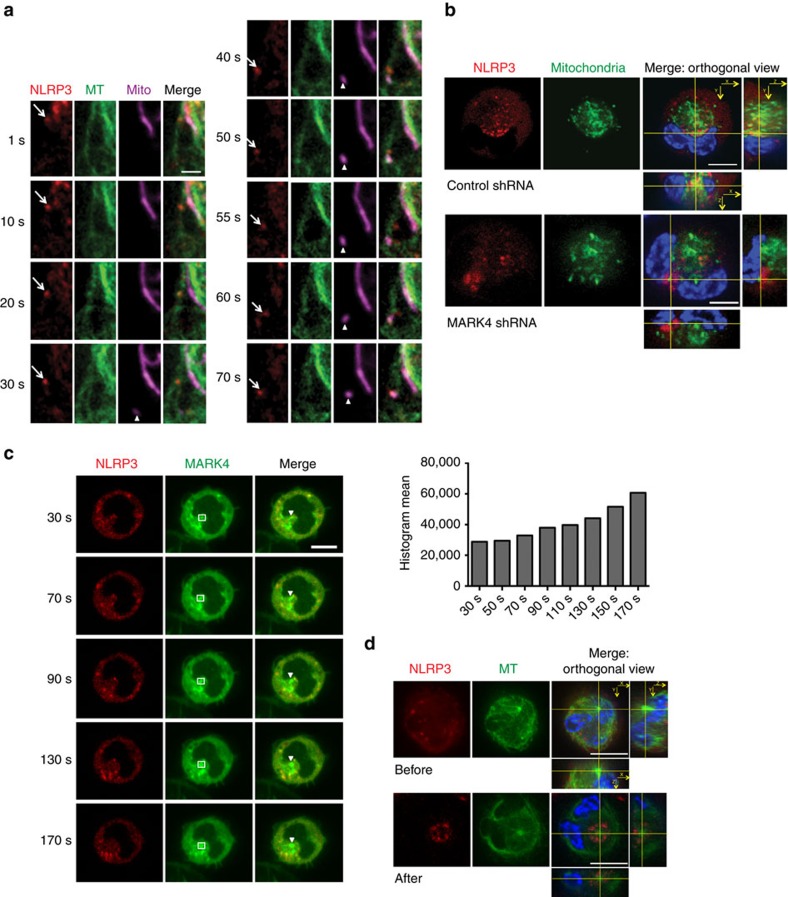
MARK4 is involved in NLRP3 positioning along microtubules. (**a**) Upon nigericin stimulation (3 μM), NLRP3 (arrow) was moving along microtubules (MT) to meet mitochondria (arrowhead) in THP-1 cells stably expressing NLRP3-cherry. Scale bar, 2 μm. See also Supplementary Movie 1. (**b**) Orthogonal view of NLRP3 with mitochondria in THP-1 cells stably expressing shRNA of MARK4 or scrambled controls. See also Supplementary Movie 2. (**c**) Cherry-NLRP3 and GFP-MARK4 were moving together towards MTOC in differentiated THP-1 cells upon nigericin stimulation. Histogram mean of MARK4-GFP was calculated within the indicated square. Arrowheads indicate MTOC. See also Supplementary Movie 4. (**d**) Orthogonal view of NLRP3 with microtubule in THP-1 cells stably expressing NLRP3-Cherry before or after stimulation by nigericin (10 μΜ for 2 h); orthogonal view was centered around MTOC. See also Supplementary Movie 5. Experiments were repeated at least three times. Scale bar, 10 μm (**b**–**d**).

A role for mitochondria in assembly of NLRP3 inflammasome has been demonstrated^6^,^27^,^28^, but we have very little understanding of how NLRP3 is mobilized from the cytosol to the mitochondria. A well-known function of MARK4 *Drosophila* homologue partitioning defective gene 1 (Par-1) is to position macromolecules like STAUFEN protein and *oskar* mRNA to the correct destination in a microtubule-dependent manner^29^. However, the mechanism through which Par-1 delivers such function is still not fully understood. We directly observed the transport of NLRP3 along microtubules in living cells. We found that stimuli causing NLRP3 inflammasome activation like nigericin, acting as polarizing inputs, induced dynamic NLRP3 transport along microtubules to meet mitochondria (Fig. 4a; Supplementary Movie 1). NLRP3 particles were able to move for a long distance (up to 12.7 μm) with a high speed (up to 577 nm s^−s^), and displayed both uni-directional and bi-directional motor-dependent movements (Supplementary Fig. 9a; Supplementary Movie 2), further supporting a microtubule-dependent transport of NLRP3. In addition, we further confirmed that shuttling of NLRP3 protein to the mitochondria was substantially reduced in MARK4 knock-down macrophages (Fig. 4b; Supplementary Movie 3; Supplementary Fig. 10), supporting a direct role for MARK4 in the transport of NLRP3 from other compartments to mitochondria. Interestingly, there was no apparent difference of run length and run speed of mobile NLRP3 particles in MARK4 knock-down cells when compared with controls (Supplementary Fig. 9b; Supplementary Movie 2), indicating that the positioning defect was not due to a delayed transport process in the absence of MARK4. Together, our results strongly support an evolutionarily conserved role of MARK4 in microtubule-dependent transport.

### MARK4 drives NLRP3 to MTOC upon inflammasome activation

MARKs were originally identified through their ability to regulate microtubule stability by phosphorylating and dissociating MAPs^30^. This association has been shown to regulate microtubule-based transport by removing MAPs that would otherwise hinder the attachment and/or movement of motors along microtubules in neuronal cells^30^. MAPs in RAW264.7 macrophages may be dynamically regulated upon inflammatory activation^31^. In addition, assembly of the NLRP3 inflammasome is regulated by acetylated α-tubulin^6^. Here we show that loss of Mark4 doesn’t affect Maps, acetylated tubulin or total tubulin levels (Supplementary Fig. 3), mitigating the possibility that aberrant NLRP3 positioning was caused by these microtubule-relevant mechanisms. MARK4 co-localizes with microtubules, and specifically binds to MTOC^10^. We found that upon inflammasome activation, MARK4 and NLRP3 moved together towards MTOC, and MARK4 was accumulating on MTOC with time (Fig. 4c; Supplementary Movie 4). By highlighting microtubule structures, we further demonstrated that NLRP3 accumulated around MTOC upon inflammasome activation (Fig. 4d; Supplementary Movie 5).

**Figure 5:**
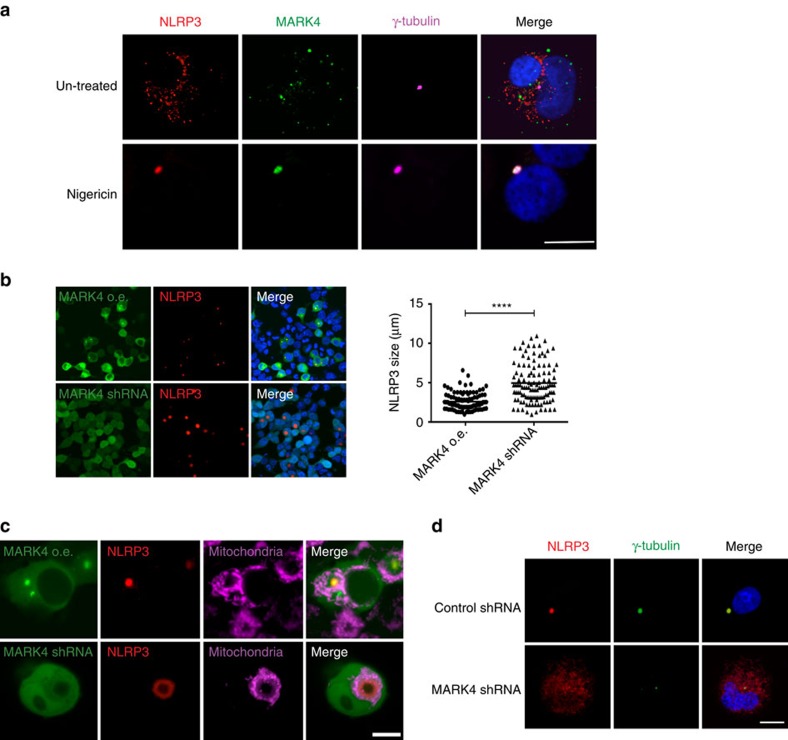
MARK4 deficiency affects MTOC and speck nucleation. (**a**) Differentiated THP-1 cells were stimulated with nigericin (10 μΜ for 1.5 h) or left untreated (control). Endogeneous levels of NLRP3 and MARK4 were co-stained with γ-tubulin. (**b**,**c**) HEK293T cells were co-overexpressed with GFP-MARK4, Cherry-NLRP3 and ASC-Flag (indicated as MARK4 GFP o.e.); or co-overexpressed with MARK4 shRNA (shown by green GFP), Cherry-NLRP3 and ASC-Flag (indicated as MARK4 shRNA). co-overexpression with MARK4-GFP drove NLRP3 to MTOC, and knock down of MARK4 by shRNA (indicated by GFP) led to a dilated ring structure of NLRP3. Quantification of speck size was shown. Scale bar, 40 μm. Mean±s.e.m. for all the cells taken from five different views at × 20 magnification for each group. Comparisons of the two different groups were analysed by unpaired *t*-test. ****P<0.0001 was considered as statistically significant (**b**). Mitochondria distribution was shown after speck formation (**c**). See also Supplementary Movie 6. (**d**) In differentiated THP-1 cells, upon nigericin stimulation (10 μΜ for 1.5 h), NLRP3 was translocated to MTOC, indicated by γ-tubulin. NLRP3 remained in cytoplasm distribution in MARK4 shRNA cell. Experiments were repeated at least three times. Scale bar, 10 μm.

It is known that inflammasome activation can lead to one big speck structure per cell^32^,^33^, but it is not known what this structure is, and why it is only a single speck complex within a given cell. Assembly of inflammasome in a unified manner is suggested to promote proximity-induced cleavage and further activation^32^, and MTOC can provide such a platform for efficient processing of inflammasome complex. Here we show that in macrophages expressing endogenous levels of MARK4 and NLRP3, MARK4 and NLRP3 co-localized on MTOC (γ-tubulin staining) and formed the classic single speck structure upon inflammasome activation (Fig. 5a). Almost every NLRP3 speck (98%) spotted had clear MARK4 co-localized with it on the MTOC. In HEK293T cells, overexpressed MARK4 drove over-expressed NLRP3 onto this speck structure on MTOC (Fig. 5b and Supplementary Fig. 11). Remarkably, MARK4 knock-down led to a dilated NLRP3 structure of 4.94 μm on average, when compared with a condensed NLRP3 speck structure of 2.59 μm on average in MARK4 overexpressed cells (Fig. 5b).

We further observed the speck structure at higher magnification, and found that NLRP3 was no longer located at mitochondria organelle after speck formation, and mitochondria were detected around the speck (Fig. 5c). Remarkably, MARK4 knock-down prevented NLRP3 from forming stereotypical speck structure on the MTOC (Fig. 5c,d). It led to a dilated NLRP3 ring structure, prevented NLRP3 nucleation on MTOC, and disrupted speck formation in HEK293T cell (Fig. 5c; Supplementary Movie 6). Disrupted speck formation and microtubule nucleation were also confirmed when endogenous levels of MAKR4 were knocked down in THP-1 cells (Fig. 5d). The remarkable prevention of NLRP3 forming speck on MTOC when MARK4 level is reduced strongly supports a critical role for MARK4 as a driving force for NLRP3 to localize and nucleate on MTOC. In addition, ASC is confirmed in the speck structure at the MTOC (Supplementary Fig. 12a). In the MARK4 knock-known cells, ASC was also dilated and not at the MTOC (Supplementary Fig. 12b), suggesting that ASC localization on the MTOC first requires NLRP3 positioning at MTOC. Moreover, we found that ASC oligomerization was reduced in MARK4 deficient cells following inflammasome activation (Supplementary Fig. 13). The effect of MARK4 on ASC oligomerization is probably indirect and may have resulted from altered NLRP3 positioning and subsequent ineffective speck formation. However, the subsided co-localization of NLRP3 and ASC in the wrong place (outside of MTOC) may still be able to constitute a partial activation, shown by compromised and incomplete IL-1β release in the MARK4 knock-out cells (Fig. 1). In summary, our data identify a previously unsuspected role for MARK4 in driving NLRP3 in the formation of one big condensed speck structure in the cell following its activation, and support an evolutionarily conserved role of MARK in the organization of microtubules and in arranging the distribution of their binding partners.

### Disruption of MARK4-NLRP3 interaction alters speck formation

**Figure 6:**
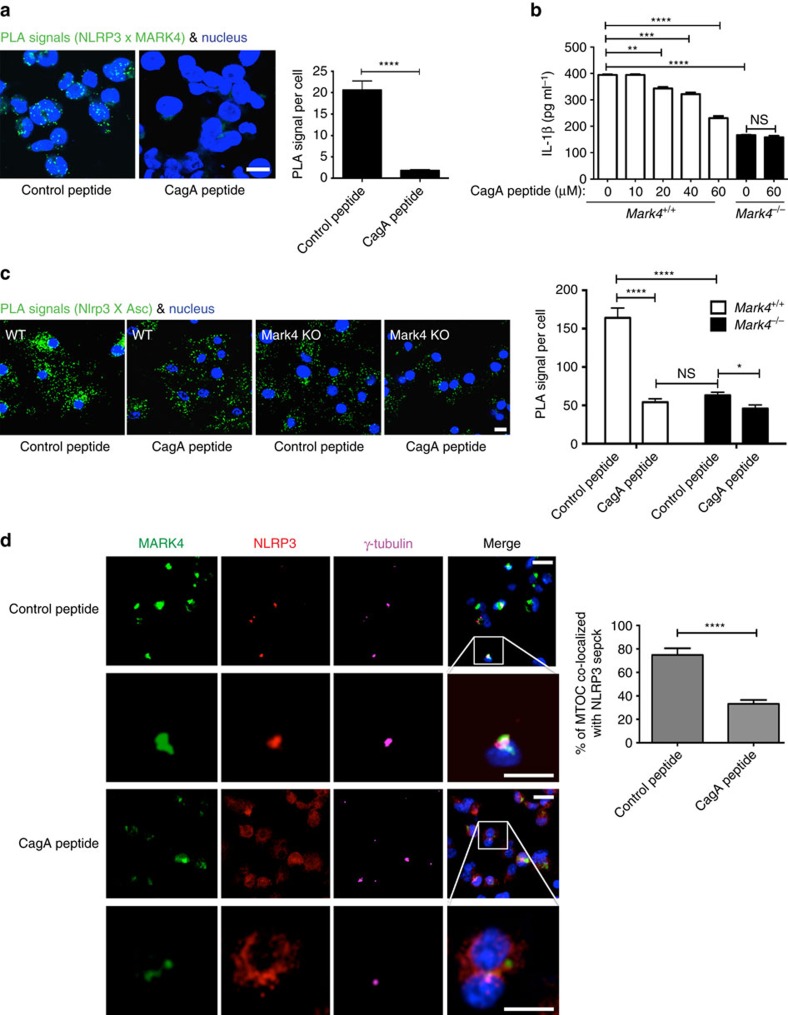
CagA impairs inflammasome activation by disrupting NLRP3-MARK4 interaction. (**a**) CagA peptide impaired the interaction between NLRP3 and MARK4 upon NLRP3 inflammasome activation by nigericin (3 μM for 2 h) in the differentiated THP-1 cells. A control peptide was employed. Scale bar, 10 μm. (**b**) ELISA of IL-1β level in the supernatants of WT BMDM cells following pretreatment of CagA peptide (with indicated concentrations) and nigericin stimulation (10 μM for 1 h). Mark4 KO cells were used as controls. (**c**) CagA peptide prevented formation of Nlrp3 and Asc complex under nigericin stimulation (3 μM for 2 h) in WT BMDMs. Mark4 KO cells were used as controls. Scale bar, 10 μm. Mean±s.e.m. for all the cells taken from five different views at × 40 magnification for each group (**a**,**c**). (**d**) CagA peptide impaired the formation of NLRP3 speck on MTOC upon NLRP3 inflammasome activation (MSU 250 μg ml^−1^ for 6 h) in the differentiated THP-1 cells. Inset was used to display magnification of the boxed region as examples of MARK4, NLRP3, and γ-tubulin localization. Scale bar, 20 μm. Mean±s.e.m., at least three experiments (**a**–**d**). Comparisons of the two different groups were analysed by unpaired *t* test. NS was considered as not statistically significant. *P<0.05, **P<0.01, ***P<0.001 and ****P<0.0001 were considered as statistically significant (**a**–**d**).

We found that a region present in NLRP3 PYD domain^34^,^35^, displayed high structural similarity with a known structure of CagA^36^ (Supplementary Fig. 14a,b), a peptide originally derived from *Helicobacter pylori* and subsequently shown to mimic the substrates of MARK proteins^15^,^18^,^36^,^37^. According to the structural alignment between NLRP3 PYD and CagA peptide, we hypothesized that CagA peptide may be employed as a NLRP3 binding competitor against MARK4 to disrupt MARK4-NLRP3 interaction. A control peptide was designed by mutating the predicted binding sites to glycine (Supplementary Fig. 14c). Indeed, incubation with CagA peptide before NLRP3 stimulation with nigericin abrogated the interaction between NLRP3 and MARK4 in THP-1 cells (Fig. 6a), confirming our hypothesis. Moreover, treatment with CagA peptide led to a dose-dependent reduction of caspase-1 activation and IL-1β production in WT BMDMs (Fig. 6b; Supplementary Fig. 14d). CagA peptide did not alter inflammasome-induced IL-1β production in Mark4 deficient cells (Fig. 6b), indicating a Mark4-dependent effect of this peptide. The results strongly support the use of CagA peptide as a potent and selective inhibitor of NLRP3 inflammasome pathway. Inhibition of Mark4/Nlrp3 interaction, by CagA treatment or using Mark4 KO, also reduced the interaction between Nlrp3 and Asc (Fig. 6c), demonstrating an important role of Mark4/Nlrp3 interaction in the assembly of an optimal inflammasome complex. Moreover, CagA peptide prevented NLRP3 from forming a speck on MTOC (Fig. 6d). The overlapping of MTOC with NLRP3 speck structure was significantly reduced by treatment with CagA peptide (Fig. 6d). Our data collectively highlight an essential role of MARK4 in generating an optimal level of NLRP3 inflammasome activation.

### MARK4 promotes NLRP3-dependent responses *in vivo*

**Figure 7:**
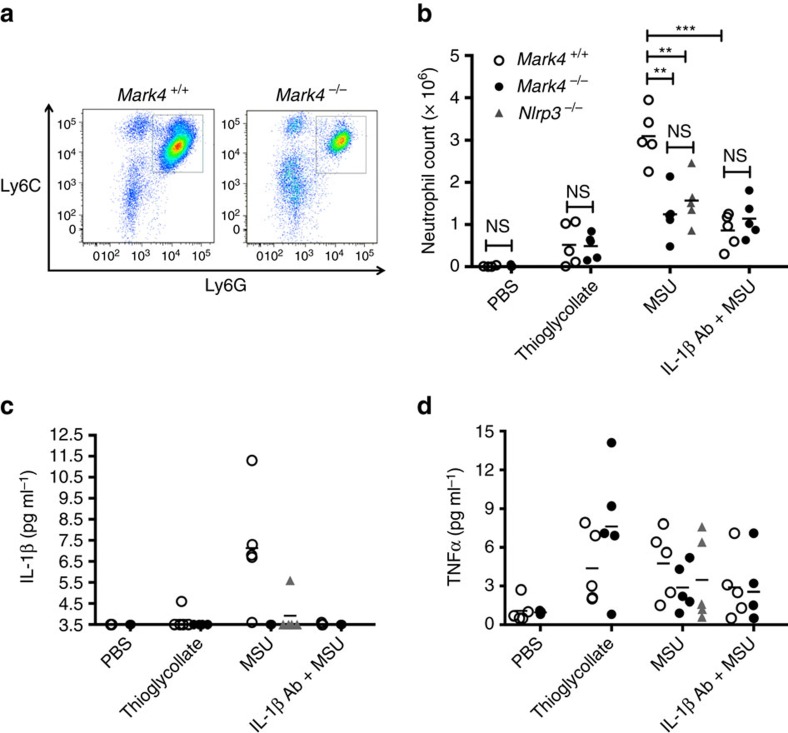
Mark4 promotes Nlrp3-dependent responses *in vivo*. Representative images of neutrophil recruitment by flow cytometry are shown in mice injected intraperitoneally (i.p.) with MSU (**a**). Neutrophil count (**b**), IL-1β (**c**) and TNF levels (**d**) in peritoneal exudate of mice (*Mark4*^−/−^, *Mark4*^+/+^, *Nlrp3*^−/−^) injected i.p. with MSU, with or without IL-1β blocking antibody. IL-1β below the detection level of 3.5 pg ml^−1^ was plotted along *Y* axis at 3.5 pg ml^−1^. Thioglycollate was used as an inflammasome-independent inflammatory agent. Mean±s.e.m., *n*=5 sex and age-matched mice (males, 8 to 10 week-old) in each group. Comparisons of the two different groups were analysed by unpaired *t* test. NS was considered as not statistically significant. **P<0.01 and ***P<0.001 were considered as statistically significant (**b**).

MSU crystals accumulate in gout disease and trigger NLRP3-dependent innate immune system activation leading to the release of IL-1β, which is responsible for the neutrophilic gouty inflammation^38^,^39^. To assess the contribution of Mark4 to Nlrp3 activation *in vivo*, we used a well-validated Nlrp3-dependent acute sterile inflammatory model in mice, induced by the injection of MSU into the peritoneal cavity^5^. As expected, we detected a pronounced influx of neutrophils (Ly6G^hi^ Ly6C^+^ cells) into the peritoneum of wild-type *Mark4*^*+/+*^ mice 6 h after MSU injection (Fig. 7a–c), which was largely dependent on NLRP3 activation and IL-1β production (Fig. 7b,c) because it was substantially reduced in *Nlrp3*^−/−^ mice or after administration of a neutralizing anti-IL-1β antibody (Fig. 7b,c). Interestingly, IL-1β production and neutrophil accumulation in peritoneal lavage were markedly reduced after MSU injection into *Mark4*^−/−^ mice compared with wild-type littermate controls (Fig. 7b,c), whereas TNF release was comparable between the 2 groups of mice (Fig. 7d), indicating a substantial and specific reduction of the inflammatory response. Furthermore, the effect of Mark4 on neutrophil accumulation was totally dependent on IL-1β production, as there was no further reduction of neutrophil recruitment in *MarK4*^−/−^ mice after treatment with IL-1β neutralizing antibody (Fig. 7b). Mark4 deletion did not affect the peritoneal inflammatory response to thioglycollate, an inflammasome-independent stimulus (Fig. 7b–d). In addition, Mark4 deletion did not compromise neutrophil migration *per se* (Supplementary Fig. 15). Thus, Mark4 was required for Nlrp3-mediated IL-1β production and inflammation *in vivo*.

## Discussion

The importance of microtubules in the response of macrophages to inflammatory stimuli has been suggested in very early studies^40^, and recent work has linked microtubule function with inflammasome activity^5^,^6^. However, the molecular mechanisms behind this function remained largely unknown. Here we identify MARK4 as a specific immune modulator of NLRP3 inflammasome. We have made several observations that point to MARK4 as a specific player in NLRP3 inflammasome pathway. First, MARK4 specifically regulates NLRP3 inflammasome but does not affect several other inflammasome pathways examined, which is rendered possibly by a unique interaction between NLRP3 and MARK4 proteins. Secondly, other MARK family members (MARK1, MARK2 and MARK3) show very limited binding ability to NLRP3, supporting a distinct role of MARK4 in this pathway. Thirdly, MARK4 regulates NLRP3 inflammasome activation by specifically targeting a fraction of NLRP3. This fraction of NLRP3 requires MARK4 for its shuttling to sites of optimal activation. Therefore, inflammasome activation is significantly reduced but not completely abolished in MARK4 knock-out cells upon both crystalline and noncrystalline activators, which may preserve a homeostatic inflammatory response. In this regard, it is interesting to note that loss of MAVS, a mitochondria protein required for assembly of inflammasome complex on this organelle, also results in a partial repression of inflammasome activation in response to non-crystalline activators, and does not alter NLRP3 inflammasome activation by non-crystalline activators^27^,^41^. Thus, it is plausible that different molecules like MARK4 and MAVS are allocated different but complementary tasks during the process of inflammasome activation, and that such division of labour may ensure optimal regulation of NLRP3 positioning and activation in response to polarizing cues. This hypothesis merits further investigation.

MARKs and their invertebrate homologue Par-1 are conserved regulators of the organization of microtubule arrays, and they are important for the establishment of cell polarity and for fate determination in polarized structures like *Drosophila* oocytes and epithelia^15^,^29^,^42^. There is a network of cross-regulatory and feedback interactions between MARK (Par-1) protein, other polarity regulators and the cytoskeleton. Studies show that distinct microtubule-based transport mechanisms contribute to the establishment of PAR and its substrates asymmetries in different cell types^30^,^43^,^44^, and PAR also drives the proper organization of microtubules^42^,^45^. However, we did not know whether similar mechanisms were employed by MARK4 in the context of the innate immune response.

Here we demonstrate that inflammasome stimuli can act as polarizing cues to initiate dynamic NLRP3 and MARK4 transport in macrophages. How MARK4 modulates microtubule organization in these contexts remains to be explored. This modulation might involve control over microtubule stability or distribution of nucleation sites, and regulation of protein trafficking through MARK4 ability to interact with components of the cell’s apparatus. Microtubule acetylation is an important regulator of NLRP3 activation^6^. However, we did not detect any alteration of total or acetylated tubulin in MARK4 deficient cells, suggesting that MARK4 regulates NLRP3 activity through different mechanisms. Rather, we showed that MARK4 affected microtubule nucleation and the distribution of its binding partner NLRP3 on microtubule polarized minus end of MTOC, in agreement with the developmentally conserved role of MARK4. Binding of MARK4 to NLRP3 was required for NLRP3 transport to the mitochondria but also for delivering NLRP3 on MTOC, leading to the formation of a single inflammasome complex per cell and ensuring optimal activation of the inflammasome. Our study reveals a previously unappreciated mechanism through which a MARK protein can facilitate the formation of a complex structure involved in the activation of innate immunity. Recent data indicate that a centrosomal protein NEK7 is important in the regulation of NLRP3 activity^7^,^8^,^9^. Our results add more to this scenario and show that delivery of NLRP3 through a MARK4-regulated machinery to the correct position on MTOC (centrosome) is also very important in determining NLRP3 activation.

Our results show that NLRP3 is very dynamic within the cell and is transported along microtubules. Why does NLRP3 need to be transported along this cytoskeleton? Inflammasome activation requires a few components to form one big complex, and also the protease activity to cleave the pro-form proteins and then deliver the final active output. Hence, inflammasome needs platforms like MTOC for efficient complex assembly and cleavage. Thus, upon receiving stimuli (polarizing cues) at the cell periphery, NLRP3 loading on the microtubule plus end track will allow its fast trafficking along microtubules for its correct delivery to microtubule minus end at MTOC (centrosome). On the basis of our data and previous knowledge, we propose that upon stimulation, on its trafficking route to MTOC, NLRP3 is first transported along the microtubule track to meet mitochondria, perhaps for its optimal energy loading, then further transported to the microtubule track minus end MTOC for optimal assembly and efficient protein cleavage (Supplementary Fig. 16). MARK4 affects the transport of NLRP3 to those destinations, rather than affecting the assembly of the big complex. In addition, we observe that once NLRP3 reaches the MTOC and assembles into a single speck structure, it is no longer localized on the mitochondria, which are now surrounding the MTOC, suggesting that localization of NLRP3 to the mitochondria is a transient state. Furthermore, it is still not clear how IL-1β is released upon NLRP3 activation, and whether IL-1β release relies on microtubule-dependent transport and MARK4 function. Those hypotheses need to be further investigated in details. However, targeting MARK4/NLRP3 interaction or loss of MARK4 significantly affects NLRP3 inflammasome transport, positioning on MTOC, ASC oligomerization, and levels of caspase-1 and IL-1β in active forms (Figs 1 and 6; Supplementary Figs 13 and 14), strongly suggesting that interaction between MARK4 and NLRP3 is required for optimal NLRP3 function.

Taken together, our data unravel novel mechanistic roles of MARK4 in regulating NLRP3 inflammasome activation. Most current therapeutic strategies aimed at reducing NLRP3-mediated diseases involve blockade of the end-product IL-1β. Such strategies entail a number of potential undesirable effects, which are frequently associated with the suppression of a major cytokine involved in a wide range of NLRP3-dependent and -independent (patho) physiological processes. In this regard, our study clearly highlights an unconventional and more selective strategy for suppression of excessive NLRP3 inflammasome through the targeting of MARK4/NLRP3 interaction.

## Methods

### Mice

*Mark4*^−/−^ (ref. 13) and *Nlrp3*^−/−^ (ref. 5) mice were described. All mice were fully backcrossed to a C57BL/6 background. Dr Aubry Tardivel (University of Lausanne, Switzerland) provided bone marrow cells from *Nlrp3*^−/−^ mice, and Prof Clare Bryant (University of Cambridge, UK) and Prof Kate Fitzgerald (UMASS medical school, USA) provided *Nlrp3*^−/−^ mice. All studies were approved by Home Office, United Kingdom, and were performed under PPL 80/2426.

### MSU-induced peritonitis model

Age (8 to 10 weeks) and sex-matched male *Mark4*^−/−^ mice and their littermate *Mark4*^+/+^ wild-type controls were used for *in vivo* experiments. The littermates were obtained by breeding *Mark4*^−/+^ parents. Mouse fed with a chow diet was injected intravenously with 10 μg of anti-IL-1β antibody (AF-401-NA, R&D). After 30 min, mouse was injected intraperitoneally with 1 mg MSU in 0.3 ml sterile PBS. Six hours after MSU injection, animals were killed by CO_2_ asphyxiation and peritoneal exudates were collected by lavage and analysed.

### Reagents

ATP, Ultrapure LPS, colchicine, nocodazole, MDP, Phorbol 12-myristate 13-acetate (PMA), and doxycycline were obtained from Sigma. Imject Alum was obtained from Thermo. Nigericin, flagellin, poly (dA:dT) and vinblastine were obtained from Enzo. iE-DAP was obtained from Invivogen. MSU crystals and cholesterol crystals were made as described^46^,^47^: cholesterol (Sigma) dissolved in 95% ethanol (12.5 g l^−1^) was heated to 60 °C, filtered through filter paper and left at room temperature to allow crystallization; 8 g of uric acid (Sigma) was dissolved in 1,600 ml of boiling water containing 49 ml NaOH, and then PH was adjusted to 7.2, and crystals were formed by gradual cooling down. DOTAP (Roche), TransIT/LT1 (MirusBio), and TransIT/TKO (MirusBio) were used as transfection reagents. Mitotracker Red, Mitotracker Deep Red FM, TubulinTracker Green, Hoechst 33342, DAPI were obtained from Invitrogen. CytoTox 96 non-radioactive cytotoxicity assay (Promega) was used to measure lactate dehydrogenase (LDH) as an indication of cell viability following manufacturer’s instruction.

### Plasmids and molecular biology

Human NLRP3 cDNA clone was purchased from Applied Biological Materials. Full-length and truncated human NLRP3 (94–979; 1–220; 1–389; 1–574; 575–979) were subcloned into pCMV-3Tag-1. Point mutations (L41G; R43G; V52G; L54G) were created by site-directed mutagenesis. Human NLRP1, mouse NLRC4, and human AIM2 cloned into pCMV-3Tag-1 (Flag tag) were obtained from Dr John MacMicking (University of Yale, USA). Human ASC subcloned into pCA7-Flag was obtained from Dr Takechi Ichinohe and Dr Yusuke Yanagi (Kyushu University, Japan). Full-length NLRP3 was subcloned into pmCherry vector (vector backbone was obtained from Dr Christien Merrifield, CNRS, France). Full-length human MARK1, MARK2, MARK3, MARK4, and truncations of MARK4 cloned into pCMV-HA vector, and GFP-MARK4 cloned into pEGFP-C2 vector were obtained from MRC-PPU (the University of Dundee, UK). Human MARK4 shRNA, scrambled shRNA control, and lentivirus packaging plasmids were purchased (Origene). Tet-O-FUW-EGFP (Addgene) was modified to subclone GFP-MARK4 or Cherry-NLRP3 into Tet-O-FUW backbone. FUW-M2rtTA (Addgene) was used to co-express the reverse tetracycline tansactivator. NLRP3 (1-219 aa) was subcloned into pGEX-4T-3 vector to express GST fusion protein.

### Antibodies

Antibodies against NLRP3 (Adipogen, Cryo2) (Sigma, HPA012878), IL-1β (R&D systems, AF-401-NA), caspase-1 (Santa Cruz, SC-154), MAP1 (Sigma, HM-1), NEK7 (Abcam, ab133514), MAP4 (BD, 611026), α-tubulin (MBL, PM054), acetylated tubulin (Santa Cruz, SC-23950), γ-tubulin (Sigma, T5326), Tom20 (Santa Cruz, FL-145, SC-11451), ERK2 (Santa Cruz, C-14), HA tag (Santa Cruz, y-11), Flag tag (Sigma, F1804) were used for western blots. Antibodies against MARK4 (Cell Signaling, 4834) (Abcam, ab124267) (house-made by MRC-PPU, the University of Dundee, UK), NLRP3 (Sigma, HPA012878; Abcam, ab4207), ASC (Enzo, ADI-905-173) (Santa Cruz, SC-33958) were used for *in situ* proximity-ligation assay. Antibodies against markers CD11b (BIOLEGEND, 101217, 1 in 800 dilution), F4/80 (ebiosciences, 25-4801-82, 1 in 200 dilution), Ly6G (BD, 551461, 1 in 400 dilution), and Ly6C (AbD SeroTec, mca771a647, 1 in 400 dilution) were used for flow cytometry. See Supplementary Fig. 15d for gating strategy for peritoneal exudates.

### Cell culture

Bone marrow cells were dissected from femur and tibia from 6-week old male mice. Bone marrow cells were differentiated into macrophages as previously described^48^ by using 25% conditional medium from L929 cells (ATCC) enriched with macrophage colony-stimulating factor (M-CSF) for 7 to 10 days. The differentiation of BMDM was confirmed by flow cytometry with markers CD11b and F4/80. THP-1 cells (ATCC) were cultured in RPMI-1640 (Invitrogen) supplemented with 10% FBS and antibiotics. THP-1 cells were differentiated into macrophages by adding 200 nM of PMA for 48 to 72 h. HEK293T cells were cultured in DMEM (Sigma) supplemented with 10% FBS, 2 mM of L-Glutamine, 1 mM of sodium pyruvate and antibiotics. Cells were cultured at 37 °C, 5% CO_2_ in the humidified incubator.

### Treatments

Inflammasome activation was induced by the following treatments as indicated: 5 mM of ATP for 30 min; 3 μM of nigericin for 2 h; 10 μM of nigericin for 1 h; 250 μg ml^−1^ of MSU for 3 h; 250 μg ml^−1^ of cholesterol crystals for 3 h; 250 μg ml^−1^ of Alum crystals for 3 h; 1 μg ml^−1^ of flagellin for 2 h; 2 μg ml^−1^ of poly (dA:dT) for 2 h; 10 μg ml^−1^ of MDP for 18 h; 10 μg ml^−1^ of iE-DAP for 18 h. Before inflammasome activation, BMDM cells were pretreated with the following reagents: 10 μM of colchicine for 1 h; 10 μM of nocodazole for 1 h; 10 μM of vinblastine for 1 h. BMDM were primed with LPS 100 ng ml^−1^ for 6 h before inflammasome activation.

### Lentivirus transduction

Lentivirus expression plasmids (Origene) were packaged and produced by transient transfection of packaging HEK293T cells (ATCC) following manufacturer’s instruction (Origene). THP1 cells or BMDM were transduced with packaged lentivirus following manufacturer’s instruction (Origene). Stably transduced THP-1 cells were selected by GFP or Cherry fluorescence using flow sorting. Tet-on system was used as described^49^ to induce Cherry-NLRP3 or GFP-MARK4 in THP-1 cells or BMDM by adding doxycycline (2 μg ml^−1^).

### Transfection

On-target siRNAs were obtained from Dharmacon technologies (Thermo), and siRNA of non-targeting pool was used as a negative control. siRNA were transfected using TransIT TKO (Mirus Bio) as per manufactuer’s instructions. HEK293T cells were transfected with plasmids using TransIT/LT1 following manufacturer’s instruction, and the transfection efficiency was achieved more than 92%. One hour before inflammasome activation, cells were transfected with CagA peptide (GFPLKRHDGVDDLSKVG) and control peptide (GFPGKGHDGGDDGSKVG) (synthesized by Thermo Scientific or Caslo Denmark) at the indicated concentrations following manufacturer’s instruction of DOTAP reagent. Flagellin and double-stranded DNA were transfected with DOTAP reagent.

### Live cell labelling and imaging

HEK293T cells or THP-1 cells were grown on the eight-well chamber (Nunc). Cells were labeled with Mitotracker Red, Mitotracker Deep Red FM, TubulinTracker Green, and Hoechst following manufacturer’s instruction. Nucleus was revealed in blue in all the images. Live cell imaging was performed at 37 °C using confocal microscopes: Carl Zeiss LSM 700, Opterra (Bruker) or Andor Spinning Disk (Oxford Instruments). *Z*-sections were taken to reconstitute 3D structure. Images were processed and analysed using ImageJ.

### Analysis of NLRP3 motility

The intracellular translocation of fluorescent NLRP3 particles was analysed using Image processing program, ImageJ (NIH). The particles movements were tracked by generating time-space plot or kymograph. First, the region of interest (ROI) in the cell was determined for each individual particle/fluorescent spot, and line ROI is drawn across the length of the movement that is manually monitored. Kymograph was generated that represents position of the fluorescent spot through the 100 frames of images. On kymograph, *x* axis represents the distance and *y* axis represents the time. The particle movement is seen as the change in position of high intensity spot from previous frames; the immobile particle appears as a single high intensity line parallel to Y axis. Examples of kymographs illustrating unidirectional, bidirectional movements along the region of interest were presented. The run lengths and velocities were then calculated manually from the position of spots on kymographs. Run length was determined as a distance fluorescent particle travelled before stalling or reversing/changing the direction of movement. Velocity was calculated as displacement per interval time. *N*=111 (from 3 cells) for Control shRNA and *N*=136 (from 4 cells) particles tracks for MARK4 shRNA were analysed.

### *In situ* proximity-ligation assay

*In situ* proximity ligation assay (*in situ* PLA) is suitable for quantitative studies of protein expressions, and to characterize modifications and interactions of proteins^20^,^21^. The cells were cultured on eight-well chamber slide (Nunc). PLA assay was performed according to manufacturer’s instruction (Olink). Living cells treated with mitotracker Red CMXRos were fixed by 4% PFA, and then subject to PLA assay. Nucleus was revealed in blue in the images. PLA signal (indicated by symbol ‘X’) per cytoplasm of a cell were acquired using Duolink analysis software (Olink). Mitochondria overplapped with PLA signal was analysed as overlap coefficient by using Zen software.

### Coimmunoprecipitation assay

Cell lysates from HEK293T cells were collected and lysed in the triton lysis buffer: 10 mM Tris-HCl pH 8.0, 2.5 mM MgCl_2_, 5 mM EGTA pH 8.0, 0.5% Triton X-100 (w/v), 1 mM Na_3_VO_4_, 50 mM NaF and 1 tablet of protease inhibitor cocktail (Roche) per 10 ml of buffer. Anti-Flag M2 magnetic beads (Sigma) were used to isolate co-immunoprecipitated complex with Flag-tagged proteins, and Anti-HA matrix (Roche) were used to isolate co-immunoprecipitated complex with HA-tagged proteins. Beads and matrix were washed following manufacturer’s instruction.

### GST pull-down assay

The GST-tagged NLRP3 (1-219aa) was induced and expressed as described^50^. Purified GST-tagged NLRP3 (1-219 aa) was immobilized on the glutathione sepharose 4B beads (GE healthcare). Purified full-length recombinant MARK4 protein was obtained from MRC-PPU (the University of Dundee, UK), and applied to those beads. Washing steps were followed as described^50^. Samples were then subjected to western blot analysis.

### ASC oligomerization assay

Cells were lysed with TBS-triton buffer (50 mM Tris-HCL, pH 7.4, 150 mM NaCl, 0.5% Triton X-100) complemented with EDTA-free protease inhibitor (Roche). The lysates were centrifuged at 6,000 g for 15 min at 4 °C. The pellets were used as triton-insoluble fraction, and the supernatants were used as triton-soluble fraction. The triton-insoluble pellets were washed twice with TBS buffer (50 mM Tris-HCL, pH 7.4, 150 mM NaCl). The pellets were then resuspended in 300 μl TBS buffer. The resuspended pellets underwent crosslinkage for 30 min at 37 °C with 2 mM disuccinimidyl suberate (Pierce), and were then centrifuged and dissolved in LDS sample buffer for western blot.

### Western blot

Supernatant of cells stimulated with inflammasome activator was collected in serum-free media. TCA protein precipitation was performed on those supernatant to detect cleaved IL-1β and caspase-1. Western samples were separated using precast NuPAGE Novex 4–12% Bis-Tris gels (Invitrogen), and proteins were transferred onto PVDF Immobilon-FL membrane (Millipore). After antibody detections, membranes were analysed using Odyssey infrared imaging system (LICOR) or enhanced chemiluminescence (ECL, GE Healthcare) to reveal the bands. See supplementary Fig. 17 for gel source data.

### Mitochondria fractionation

BMDM cells were primed with 1 μg ml^−1^ of LPS for 4 h, then stimulated with 7.5 μM nigericin for 30 min. Subcellular fractions were obtained using mitochondria isolation kit for cultured cells (Thermo, Cat. NO. 89874), and then subject to western blot. Tom20 was used to reveal mitochondria fraction, and ERK2 was used to reveal cytoplasm fraction.

### ELISA and meso scale discovery

ELISA kits of mouse IL-1β ELISA (559603), human IL-1β ELISA (557953) were obtained from BD biosciences, and Mouse TNF ELISA (DY410) was obtained from R&D. ELISA assays were performed according manufacturer’s instructions. MSD assay of mouse IL-1β was performed by core biochemical assay laboratory of Cambridge University Hospitals, and the detection limit of mouse IL-1β by MSD is 3.5 pg ml^−1^.

### Flow cytometry

Peritoneal exudate cells were collected by lavage. Cells were stained with markers CD11b, Ly6G, and Ly6C. Stained cells were subject to Fortessa Cell Analyser (BD, USA). Ly6G^hi^ Ly6C^+^ cells from CD11b positive cells were counted as neutrophil. Data were analysed by FlowJo.

### Neutrophil migration assay

Mouse neutrophils were isolated from blood using miltenyi biotec kit (Miltenyi). Isolated neutrophil were then subject to transwell migration assay, and CXCL12 was used as attractant. The incubation was performed in 37 °C cell culture incubator for 4 h. Migrated cells were then collected and counted.

### Phagocytosis assay

Cells were co-incubated with MSU crystals for 4 h at in 37 °C cell culture incubator. Cells were then thoroughly washed and stained with Cholera Toxin Subunit B (Invitrogen). Phagocytosed MSU images were taken by reflection confocal microscopy. Fluorescence intensity of phagocytosed MSU was analysed by reflection signals per cell. Analysis was performed using ImageJ. Vybrant phagocytosis assay kit (Invitrogen) was also used following manufacturer’s instructions.

### Statistics

Statistical analysis was performed with Prism (Graphpad). All data were expressed as Mean±s.e.m. Comparisons of the two different groups were analysed by unpaired *t* test. *P*<0.05 (*), *P*<0.01 (**), *P*<0.001(***), or *P*<0.0001(****) was considered statistically significant.

### Data availability

The data that support the findings of this study are available from the corresponding author upon reasonable request.

## Additional information

**How to cite this article:** Li, X *et al*. MARK4 regulates NLRP3 positioning and inflammasome activation through a microtubule-dependent mechanism. *Nat. Commun.* **8**, 15986 doi: 10.1038/ncomms15986 (2017).

**Publisher’s note:** Springer Nature remains neutral with regard to jurisdictional claims in published maps and institutional affiliations.

## Supplementary Material

Supplementary Information

Supplementary Movie 1

Supplementary Movie 2

Supplementary Movie 3

Supplementary Movie 4

Supplementary Movie 5

Supplementary Movie 6

Peer Review File
