# Supplementary material for: MARK4 regulates NLRP3 positioning and inflammasome activation through a microtubule-dependent mechanism
Source: Nat Commun. 2017 Jun 28;8:15986. doi: 10.1038/ncomms15986 (PMC5493753; doi:10.1038/ncomms15986)
Supplement: Supplementary Information [file ncomms15986-s1.pdf]

Type of file: PDF
Title of file for HTML: Supplementary Information
Description: Supplementary Figures

Type of file: MOV
Title of file for HTML: Supplementary Movie 1
Description: NLRP3 is moving along microtubule. PMA-differentiated THP-1 cells stably expressing Cherry-NLRP3 (red) were stained with TubulinTracker green to label microtubules (green), and Mitotracker Deep Red FM to label mitochondria (magenta). Arrow indicates a NLRP3 particle, and arrowhead indicates a mitochondrion. Cells were stimulated with nigericin (3 μM). Video corresponds to 105.27 seconds; width of the movie is 23.3 μm.

Type of file: MOV
Title of file for HTML: Supplementary Movie 2
Description: Movement of NLRP3 in MARK4 knock-down cells. PMA-differentiated THP-1 cells stably expressing Cherry-NLRP3 and shRNA (scrambled control or MARK4) were stimulated with nigericin (3 μM). Video corresponds to 291 seconds; width of each movie is 25.5 μm.

Type of file: MOV
Title of file for HTML: Supplementary Movie 3
Description: Knock-down of MARK4 affects translocation of NLRP3 on mitochondria. PMA-differentiated THP-1 cells stably expressing Cherry-NLRP3 (red) and shRNA (scrambled control or MARK4) were stimulated with nigericin (3 μM for 2 hrs), and then stained with mitotracker dye to label mitochondrion (green). Z section pictures were stacked to reconstitute 3D view.

Type of file: MOV
Title of file for HTML: Supplementary Movie 4
Description: MARK4 and NLRP3 are moving together to MTOC. PMA-differentiated THP-1 cells stably expressing Cherry-NLRP3 (red) and GFPMARK4 (green) were stimulated with nigericin (10 μM). Arrowhead indicates MTOC where MARK4 is accumulated. Video corresponds to 198.366 seconds; width of the movie is 30.3 μm.

Type of file: MOV
Title of file for HTML: Supplementary Movie 5
Description: NLRP3 is shuttling towards MTOC. PMA-differentiated THP-1 cells stably expressing Cherry-NLRP3 (red) were stained with Tubulin Tracker green to label microtubules (green) before or after nigericin stimulation (10 μM for 2 hrs). Z section pictures were stacked to reconstitute 3D view.

Type of file: MOV
Title of file for HTML: Supplementary Movie 6
Description: Insufficient MARK4 causes dilated ring structure of NLRP3. HEK293T cells were co-overexpressed with GFP-MARK4, Cherry-NLRP3 and ASC-Flag (indicated as MARK4 GFP o.e.); or co-overexpressed with MARK4 shRNA (shown by green GFP), Cherry-NLRP3 and ASC-Flag (indicated as MARK4 shRNA). One day after expression, cells were subject to imaging. Z section pictures were stacked to reconstitute 3D view.

Type of file: PDF
Title of file for HTML: Peer Review File
Description:

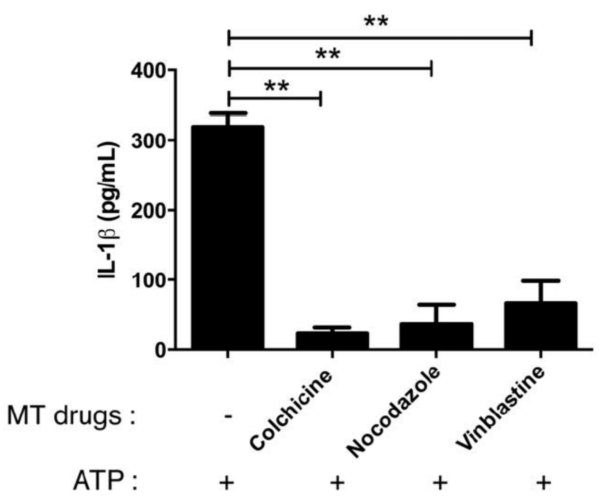

Supplementary Figure 1. Microtubule-disrupting drugs reduce IL-1β production. Microtubule disrupting drugs colchicine (10 μM), nocodazole (10 μM) and vinblastine (10 μM) were used to pretreat WT BMDM 30 minutes before ATP stimulation (5 mM) for half an hour. Supernatant was collected, and IL-1β was measured using ELISA. Mean ± SEM, three experiments. Comparisons of the two different groups were analysed by unpaired t test. $P < 0.01$ (**) were considered as statistically significant (a, b, c, d).

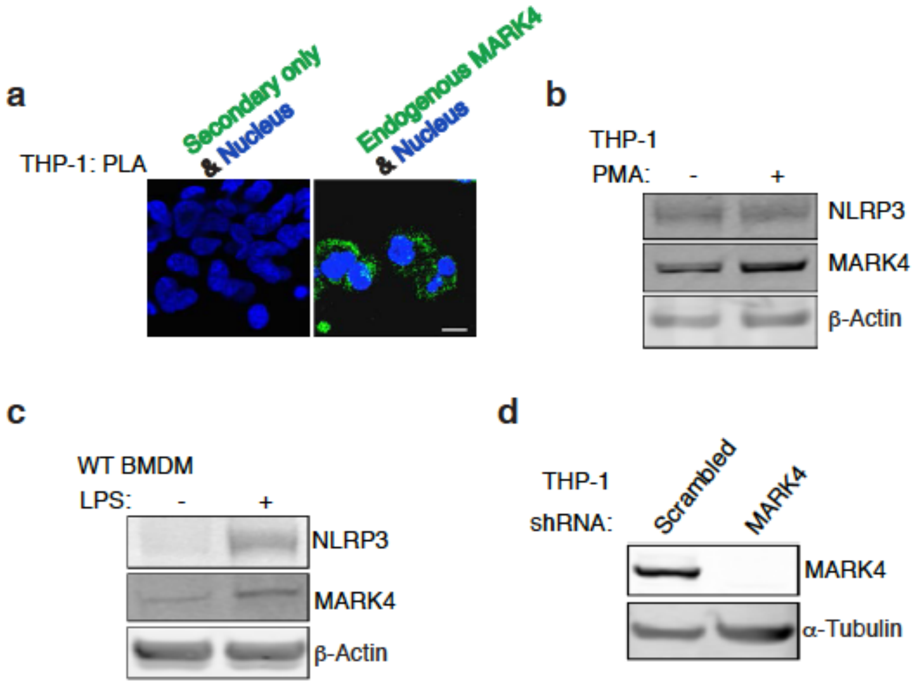

Supplementary Figure 2. MARK4 expression in macrophages. (**a**) MARK4 expression under nigericin (3 µM for 2 hrs) stimulated conditions in differentiated human THP1 cells. Scale bar = 10 µm. (**b, c**) Western blots to confirm MARK4 and NLRP3 expression in undifferentiated THP-1 monocytes or PMA-differentiated THP-1 macrophages (b), or wild type BMDM before or after LPS priming (c). (**d**) MARK4 expression in THP-1 cells after knocking-down by shRNA MARK4 or shRNA scrambled control.

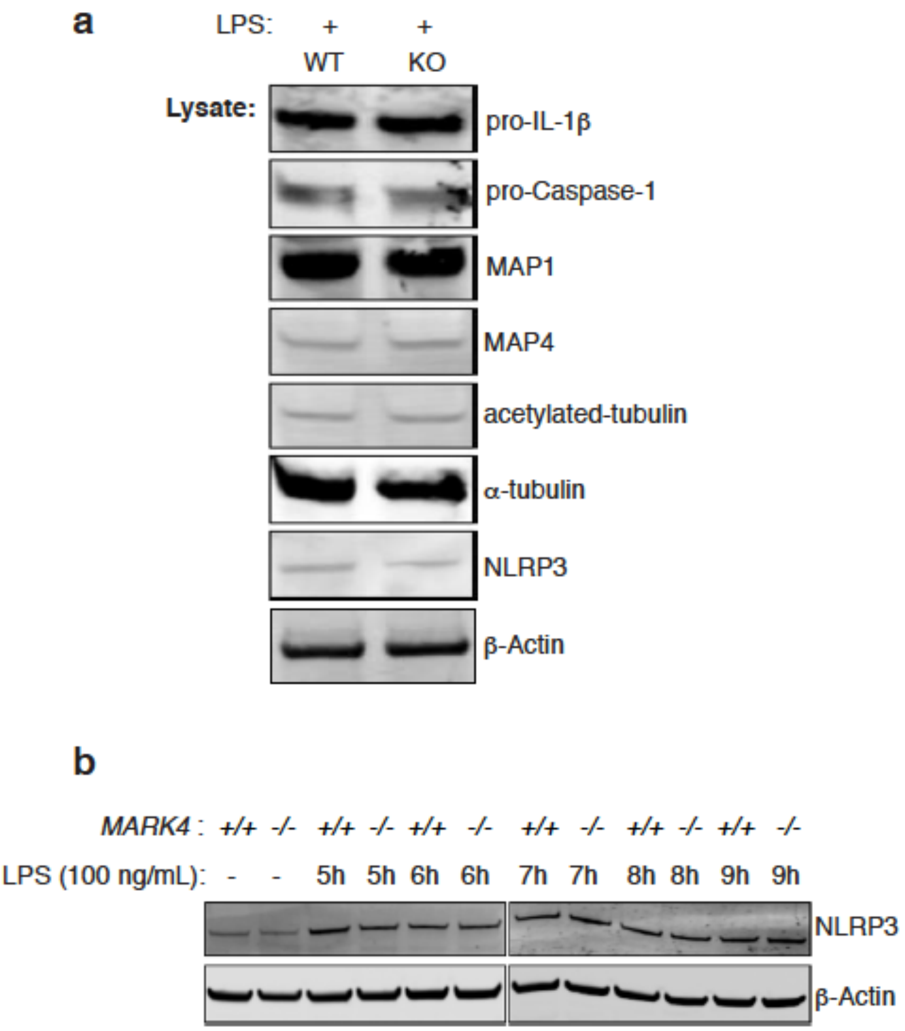

Supplementary Figure 3. The expression profile of MARK4 knock-out cells. (**a**) Western blot of cell lysates collected from BMDM of wild type or MARK4 KO mice. Cells were primed with LPS (100 ng/mL).(**b**) A time course of LPS priming (100 ng/mL) on BMDM of wild type or MARK4 KO cells. Western blot of cell lysates collected from these cells.

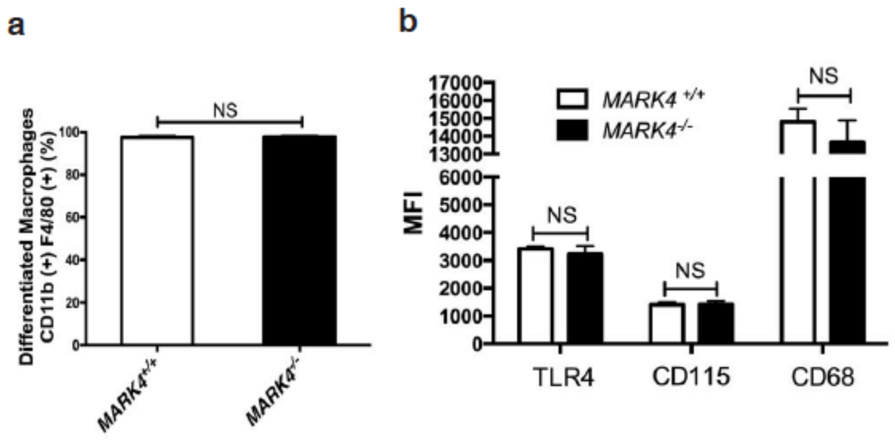

Supplementary Figure 4. The differentiation of MARK4 knock-out cells. (**a**) Flow cytometry analysis of macrophage differentiation using CD11b (+) F4/80(+) double positive markers. (**b**) LPS receptor TLR4, M- CSF receptor (CD115), and macrophage differentiation markers were compared between wild-type and MARK4 deficient BMDMs. Comparisons of the two different groups were analysed by unpaired t test. NS was considered as not statistically significant.

a

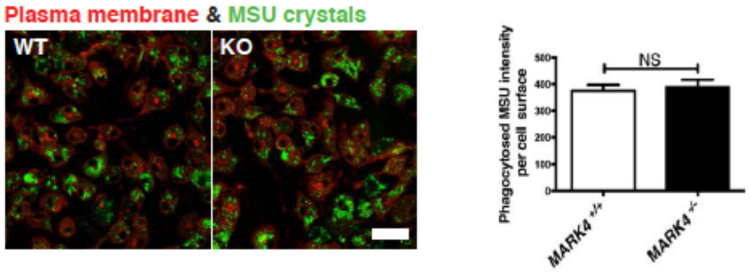

b

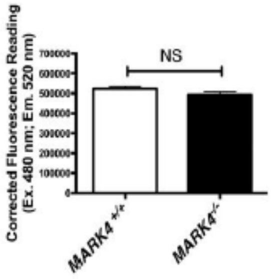

Supplementary Figure 5. Phagocytosis ability of MARK4 knockout cells. (**a**) Wild-type or MARK4 deficient BMDM were incubated with MSU crystals. Phagocytosed MSU was taken by reflection microscopy and plasma membrane was highlighted by cholera toxin staining. Scale bar= 40 µm. (**b**) Phagocytosis ability of MARK4 knockout cells was also analyzed by Vybrant phagocytosis assay following manufacturer's instruction. Comparisons of the two different groups were analysed by unpaired t test. NS was considered as not statistically significant.

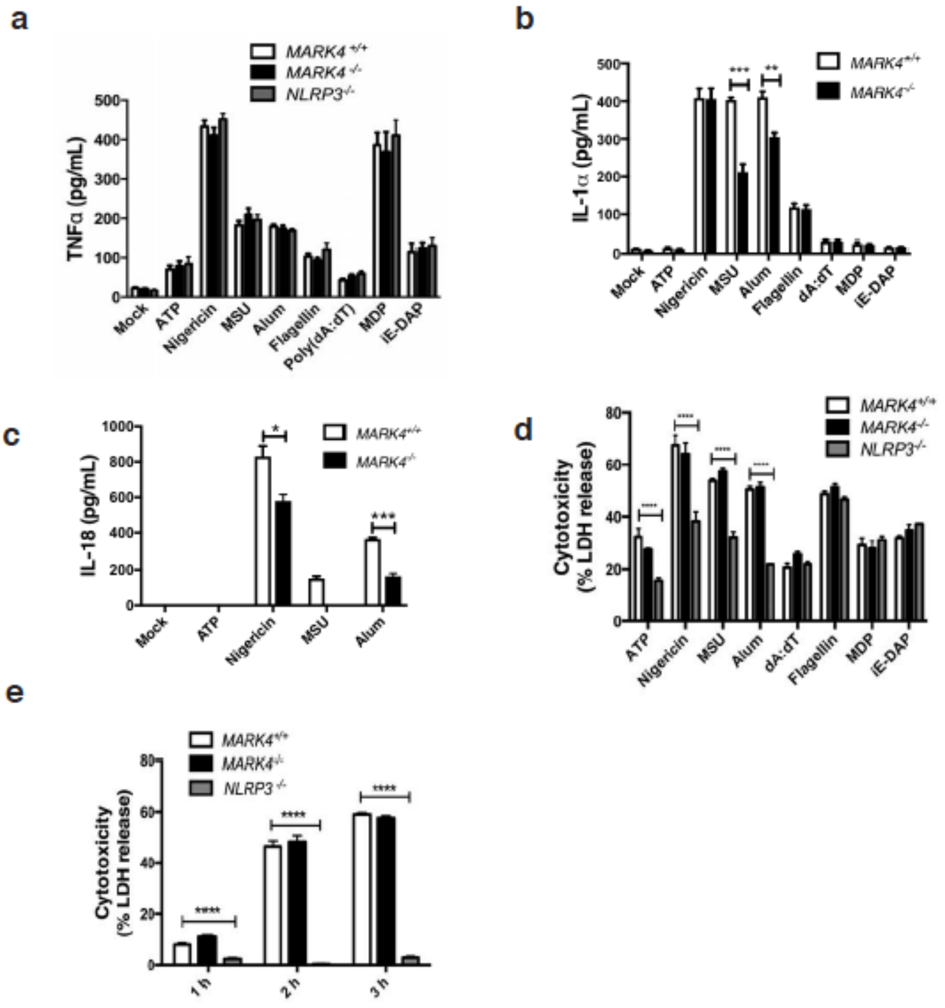

Supplementary Figure 6. The effect of stimulation on MARK4 knock-out cells. (**a, b, c,d**) BMDM derived from MARK4 WT or KO mice were treated with various stimuli indicated. TNF (a), IL-1α (b), and IL-18(c) were measured using ELISA, and cell death was measured by using LDH cytotoxicity assay (d). (**e**) Cell death induced by ATP (5 mM) over a time course was measured by LDH cytotoxicity assay. Mean ± SEM, three to four experiments. Comparisons of the two different groups were analysed by unpaired t test. $P < 0.05$ (*), $P < 0.01$ (**), $P < 0.001$(***), and $P < 0.0001$(****) were considered as statistically significant.

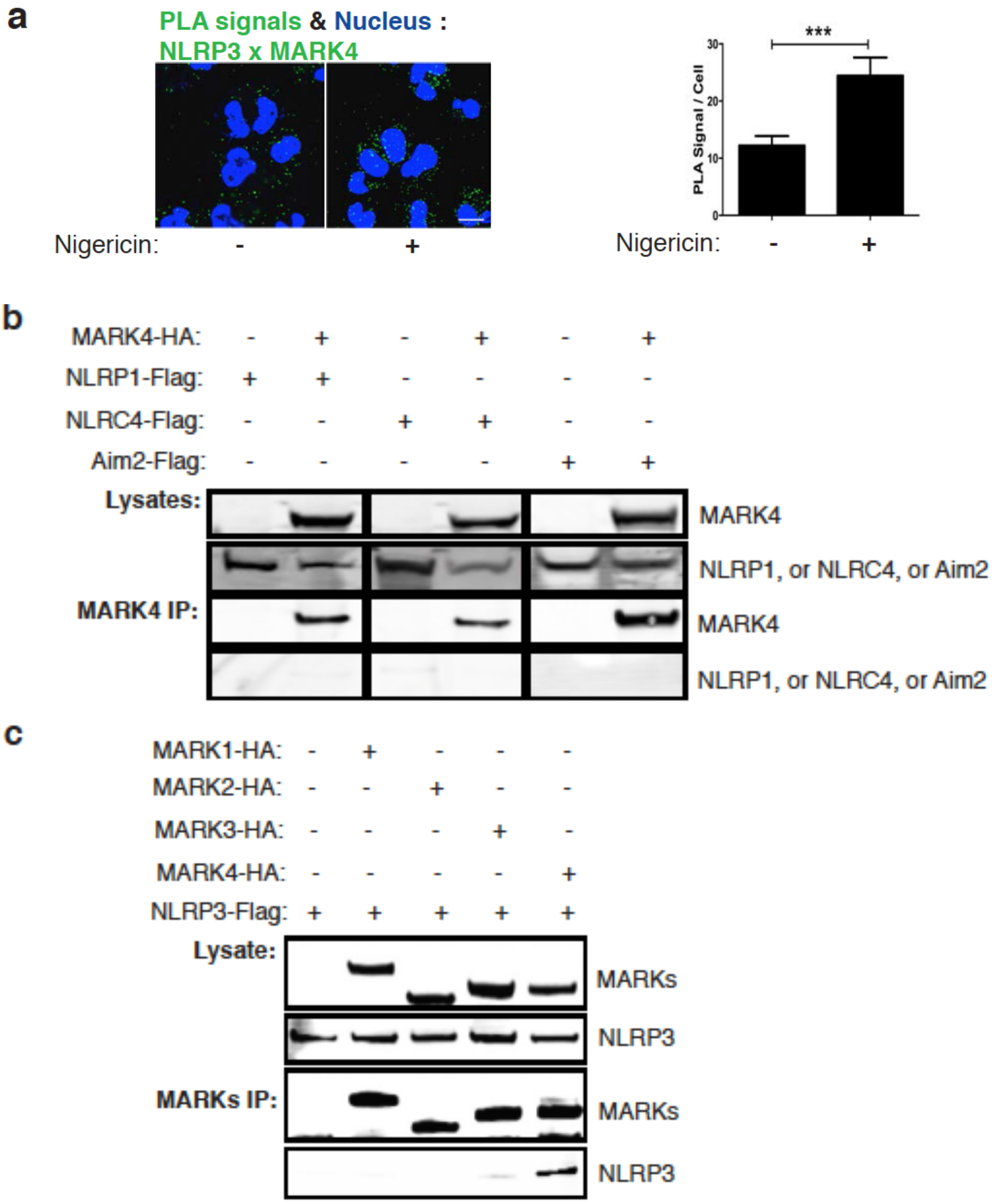

Supplementary Figure 7. MARK4 selectively binds to NLRP3. (**a**) PLA signal of NLRP3 and MARK4 in THP-1 cells, with or without nigericin stimulation (3 μM for 2 hrs). Scale bar= 10 μm. Comparisons of the two different groups were analysed by unpaired t test. $P < 0.001$(***) was considered as statistically significant. (**b**) Western blots of co- immunoprecipitated NLRP1, NLRC4, or Aim2 respectively with MARK4. (**c**) Western blots of co- immunoprecipited MARK1, 2, 3, or 4 respectively with NLRP3. Proteins were transiently co-over-expressed in HEK-293T cells. Results are representatives of three experiments.

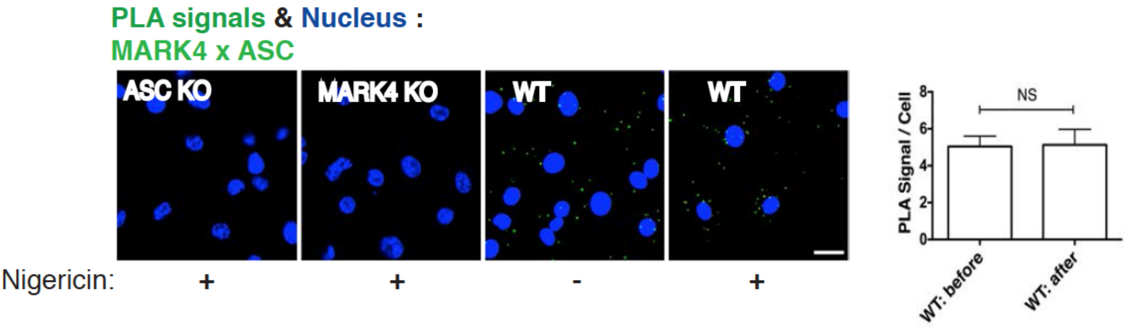

Supplementary Figure 8. Interaction of MARK4 and ASC. PLA signal of MARK4 and ASC in BMDM, with or without nigericin stimulation (3 µM for 2 hrs). Mean ± SEM for all the cells taken from at least 5 different views at 40X magnification for each group. Experiments have been repeated three times. Scale bar = 10 µm. Comparisons of the two different groups were analysed by unpaired t test. NS was considered as not statistically significant.

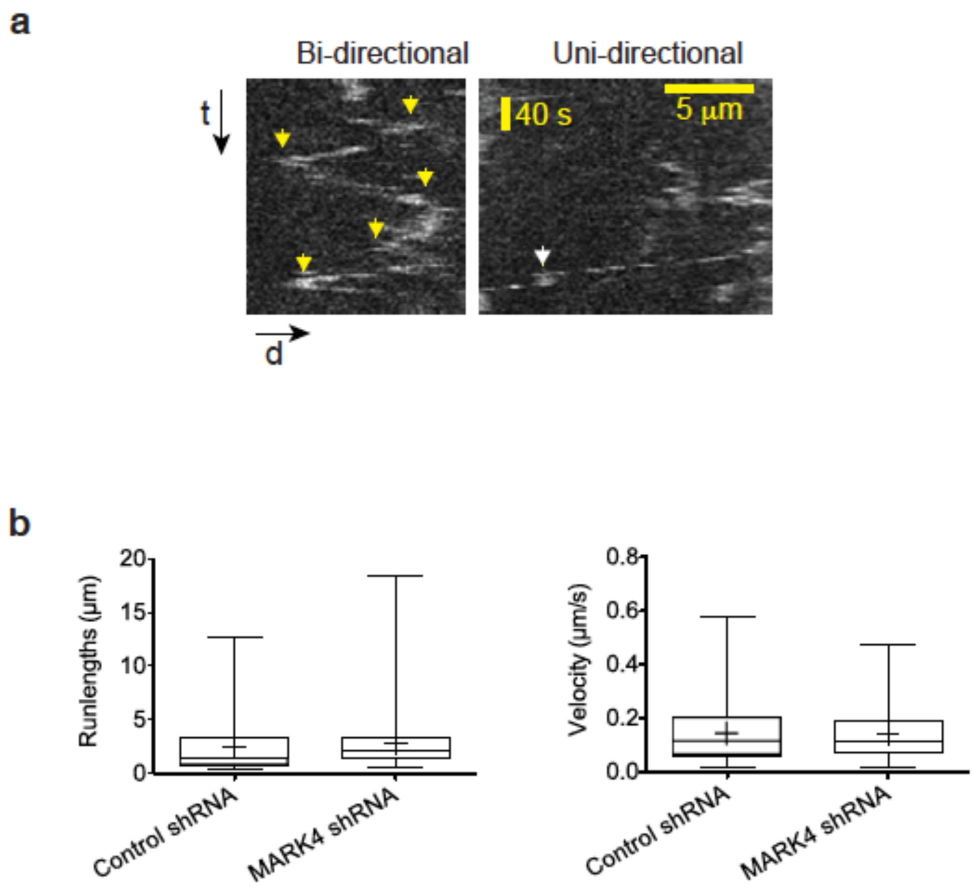

Supplementary Figure 9. Analysis of NLRP3 motility. (**a**) Representative kymographs showing uni and bidirectional movements of NLRP3 particles in differentiated THP-1 cells after stimulation with nigericin (3 µM) . It illustrates an example when fluorescent spots did not show any displacement over time. Yellow arrowheads show reversal of movement direction by an individual particle, while white arrowhead depicts 'pause' event before the particle continues to travel in the same direction. Scale bars for X (displacement in µm, 'd') and Y (time in s, 't') axis are shown as yellow lines in the kymograph. (**b**) Box and whisker plots presents dispersion of run lengths measured for the NLRP3 particle movements in Control or MARK4 shRNA background. Mean run length is illustrated by '+' sign in box and whisker plot, while horizontal line describes median. Mean run length of 2.417 ± 0.237 µm (Mean ± SEM) and 2.864 ± 0.222 µm was observed in cells with Control and MARK4 shRNA respectively. Mean velocity of 0.138 ± 0.010 µm/s and 0.142 ± 0.008 µm/s was observed in cells with Control and MARK4 shRNA respectively. See also Supplementary Movie 3.

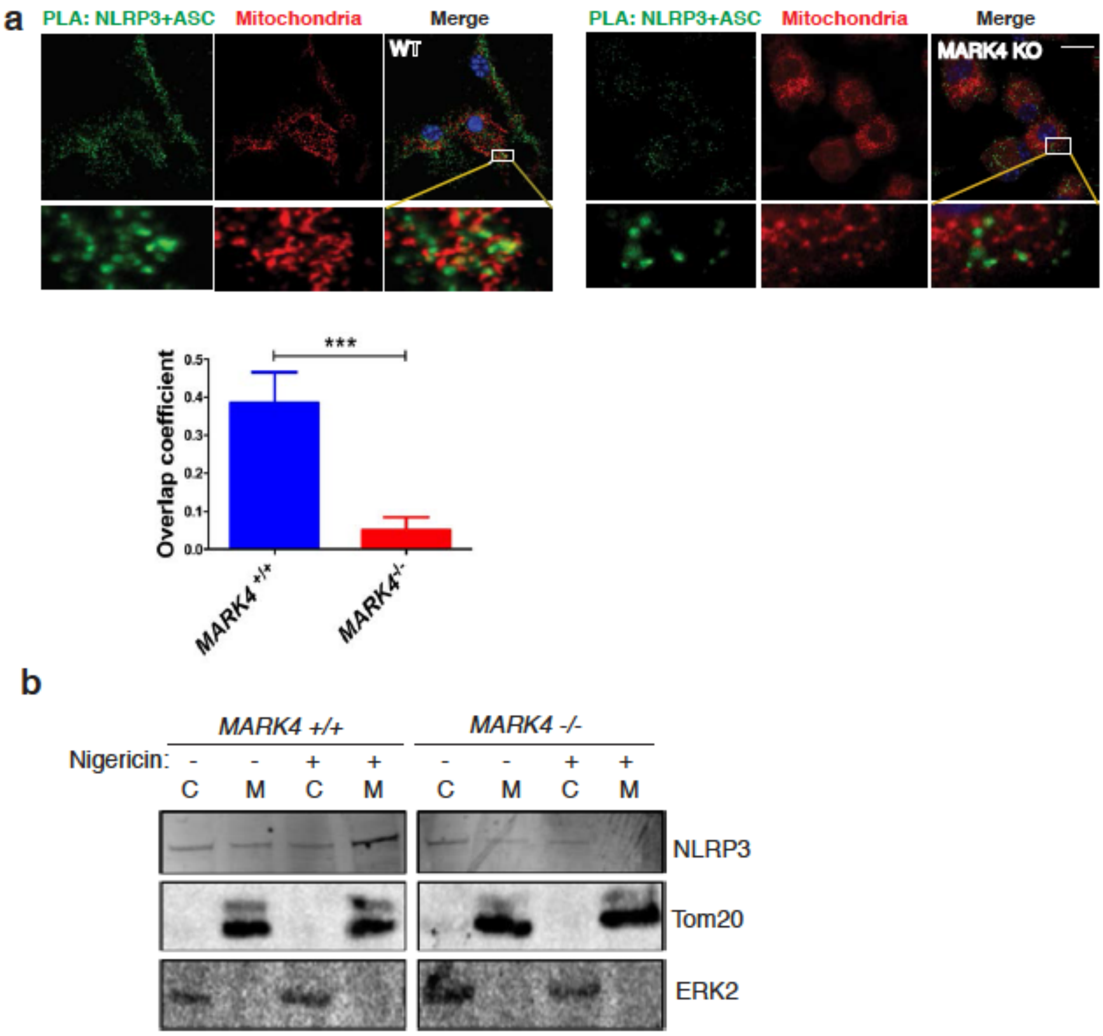

Supplementary Figure 10. MARK4 is involved in positioning NLRP3 onto mitochondria. (**a**) PLA signal of NLRP3 and ASC complex on mitochondria in WT or MARK4 KO BMDM cells after nigericin stimulation (3 µM for 2 hrs).. Overlap coefficient was shown as Mean ± SEM for all the cells taken from at least 5 different views at 40x magnification for each group. Scale bar= 10 µm. (**b**) Subcellular fractionation of WT and MARK4 KO BMDMs after nigericin stimulation (7.5 µM for 30 mins). C indicates cytosol, M indicates mitochondria. Western blots are representatives of 3 independent experiments. Comparisons of the two different groups were analysed by unpaired t test. *P* < 0.001(***) was considered as statistically significant.

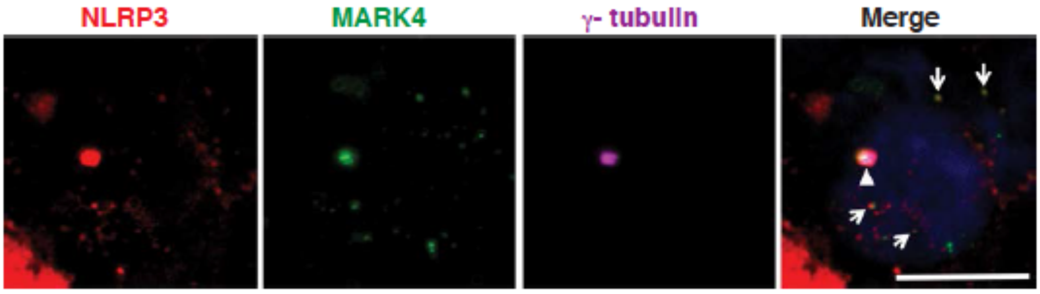

Supplementary Figure 11. MARK4 and NLRP3 are co-localized on MTOC after inflammasome activation. HEK293T cells co-overexpressing NLRP3-Cherry, MARK4-GFP, and ASC-Flag were fixed and stained with γ-tubulin. Arrowhead indicates γ-tubulin, and arrows indicate co-localized NLRP3 with MARK4. Scale bar = 10 μm.

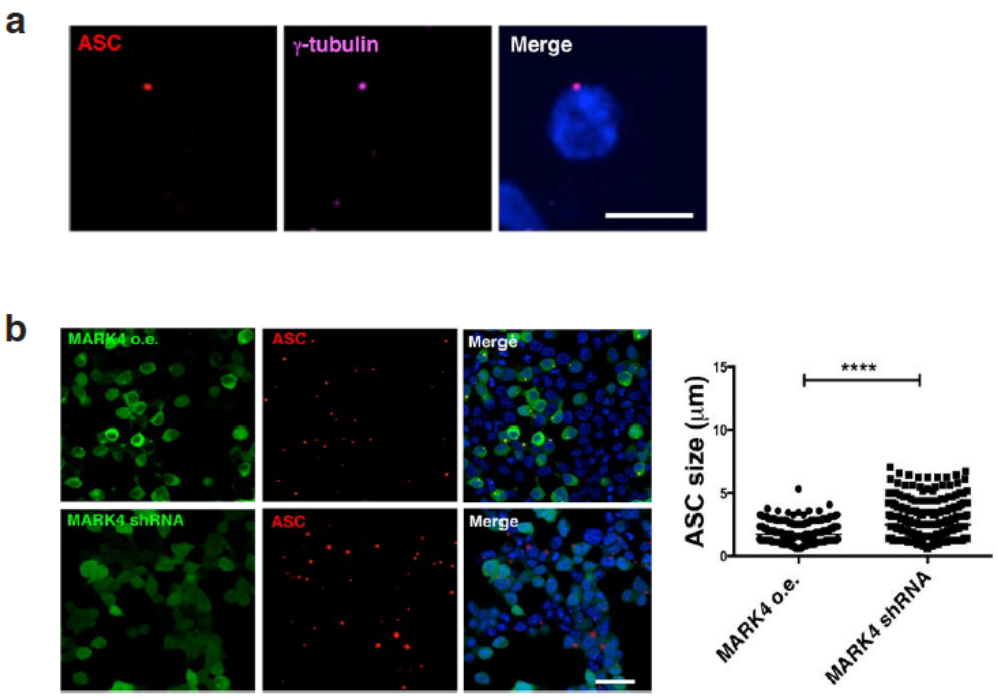

Supplementary Figure 12. ASC speck is localized on the MTOC. (**a**) Differentiated THP-1 cells were stimulated with nigericin (10 μM for 1.5 hrs) or left untreated (control). Endogeneous ASC forms speck on MTOC, indicated by γ-tubulin. Scale bar = 10 μm. (**b**) HEK293T cells were co-overexpressed with GFP-MARK4, NLRP3-Flag and Cherry-ASC (indicated as MARK4 GFP o.e.); or co- overexpressed with MARK4 shRNA (shown by green GFP), NLRP3-Flag and Cherry-ASC (indicated as MARK4 shRNA). In HEK293T cells, knock down of MARK4 by shRNA (indicated by GFP) led to an enlarged structure of ASC. Quantification of ASC speck size is shown. Scale bar = 40 μm. Comparisons of the two different groups were analysed by unpaired t test. $P < 0.0001$(****) was considered as statistically significant.

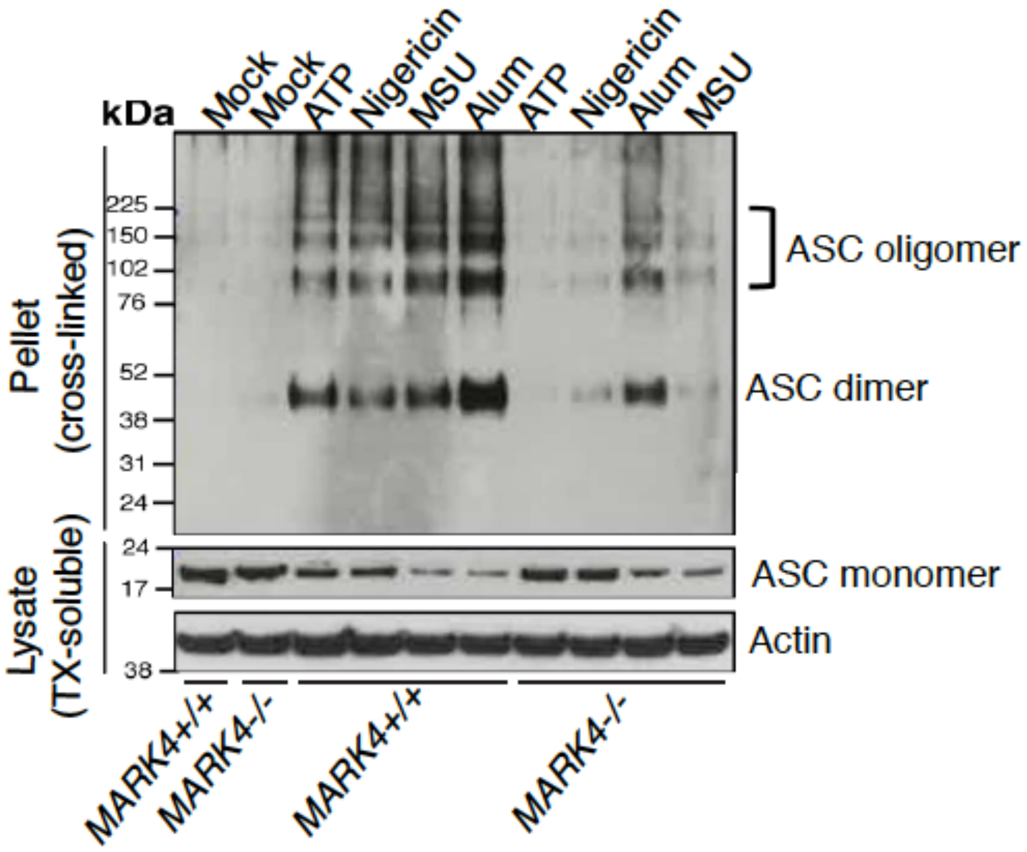

Supplementary Figure 13. ASC oligomerization is altered in MARK4 deficient cells. ASC oligomerization induced by indicated stimuli in MARK4+/+ and MARK4-/- LPS- primed BMDMs. TX, Triton X-100.

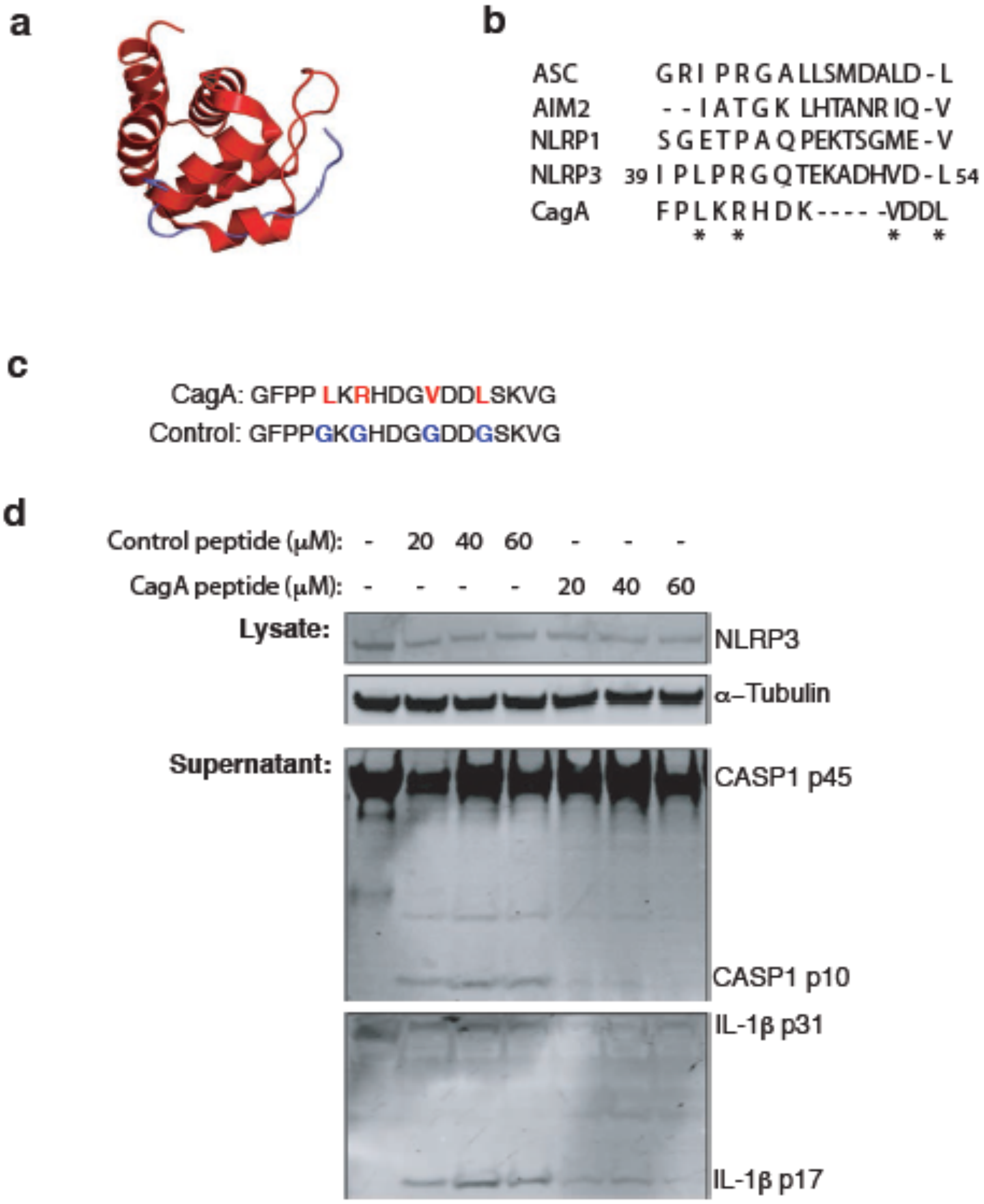

Supplementary Figure 14. CagA peptide exhibits structural similarity with pyrin domain of NLRP*3*. (**a**) Structural alignment of CagA peptide (blue) (PDB: 3IEC) with pyrin domain of NLRP*3* (PDB: 3QF2). **(b)** Alignment of pyrin domain-containing proteins NLRP**3**, NLRP**1**, ASC, And AIM*2* with CagA peptide. **(c)** Control peptides by mutating the predicted binding sites to glycine were employed. **(d)** Representative western blots of Caspase-**1** (CASP1) and IL-**1**β in the supernatant (Sup.) in CagA peptide and control peptide-treated macrophages activated with indicated stimuli. Cell lysates were used as controls to indicate equal amount of cells for analysis.

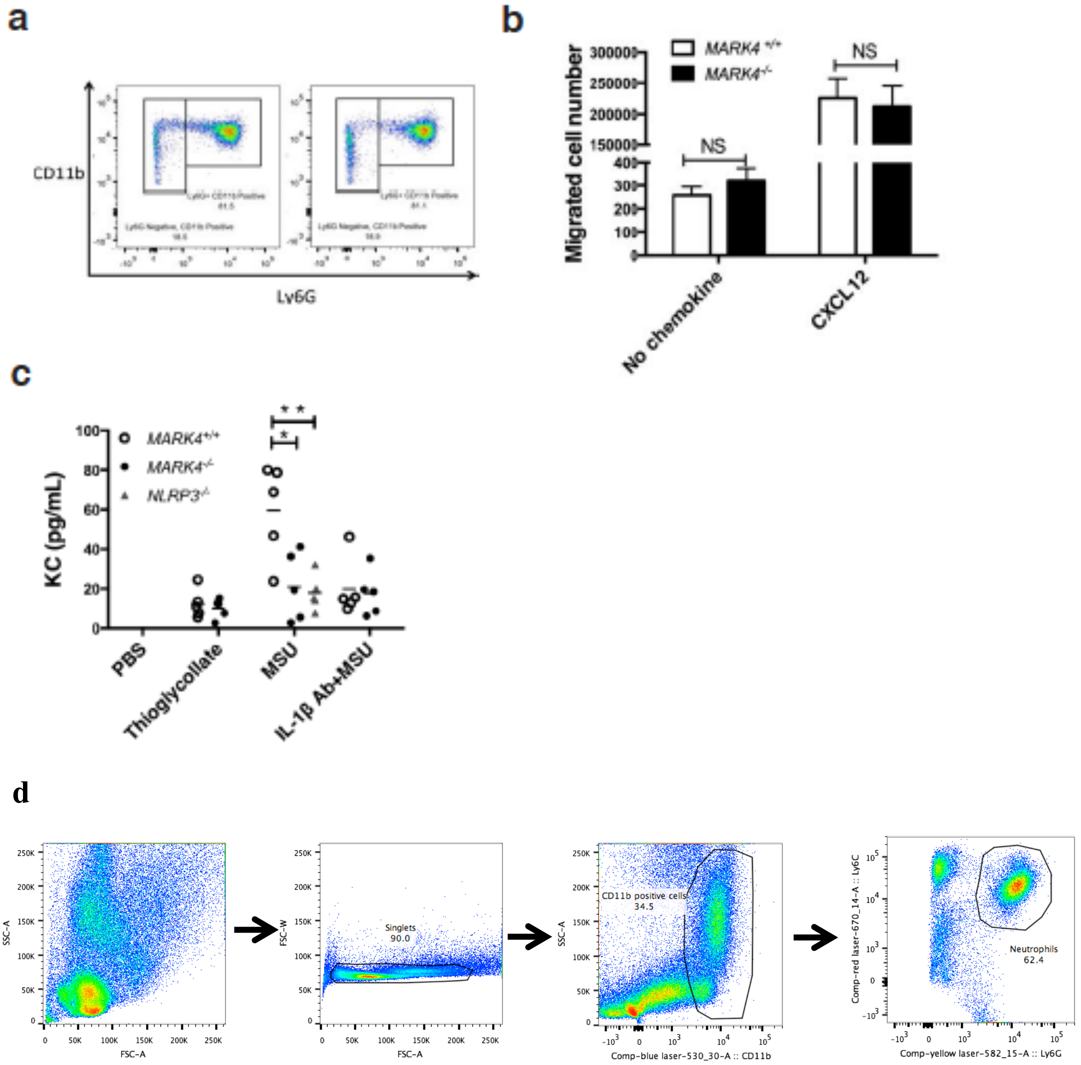

Supplementary Figure 15. The effect of MARK4 deficiency on neutrophil migration. (**a**) Mouse neutrophils isolated from blood (a) were subject to transwell migration assay. (**b**) Migrated neutrophil number was counted upon chemokine CXCL12 attraction. (**c**) KC levels in peritoneal exudate of mice (*MARK4*$^{-/-}$, *MARK4*$^{+/+}$, *NLRP3*$^{-/-}$) injected i.p. with MSU, with or without IL-1β blocking antibody. (**d**) Example of flow cytometry gating strategy for peritoneal exudates. Total cells were gated at single cells, then single cells were gated at CD11b positive cells, and then CD11b positive cells were gated at Ly6G^{high} Ly6C^{high} as neutrophils. Comparisons of the two different groups were analysed by unpaired t test. NS was considered as not statistically significant. $P < 0.05$ (*), and $P < 0.01$ (**) were considered as statistically significant.

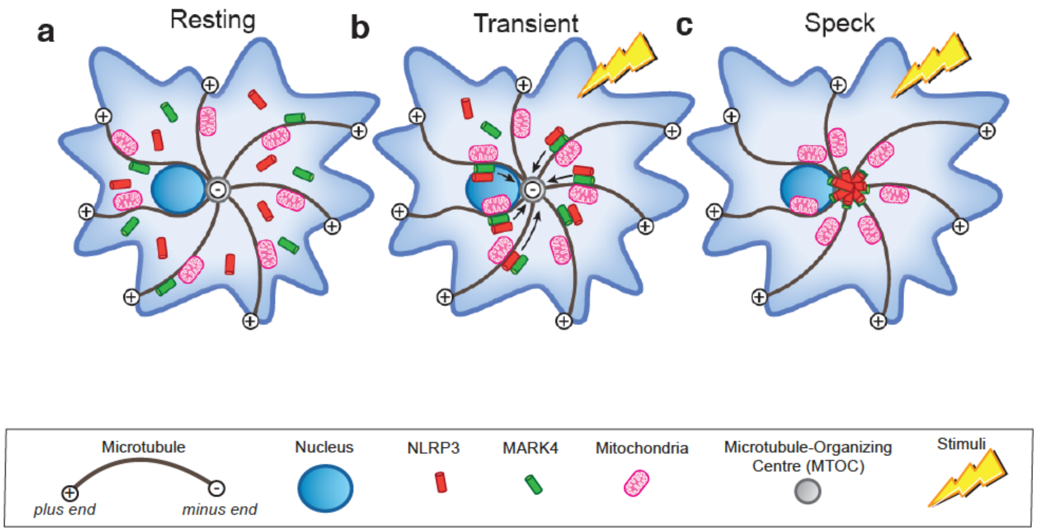

Supplementary Figure 16. Schematic representation of the role of MARK4 in positioning NLRP3. (a) In the resting state, MARK4 and NLRP3 are distributed throughout the cytoplasm. (**b**) Upon NLRP3 inflammasome stimulation, MARK4 binds to NLRP3 and transports it along microtubules to ensure optimal positioning and activation. During the transport process, they can encounter mitochondria. (**c**) Where NLRP3 may receive additional activatory cues, NLRP3 and MARK4 are further transported along microtubules to reach MTOC, where NLRP3 forms the characteristic speck structure required for optimal inflammasome activation.

Fig. 1e (Gel source data)

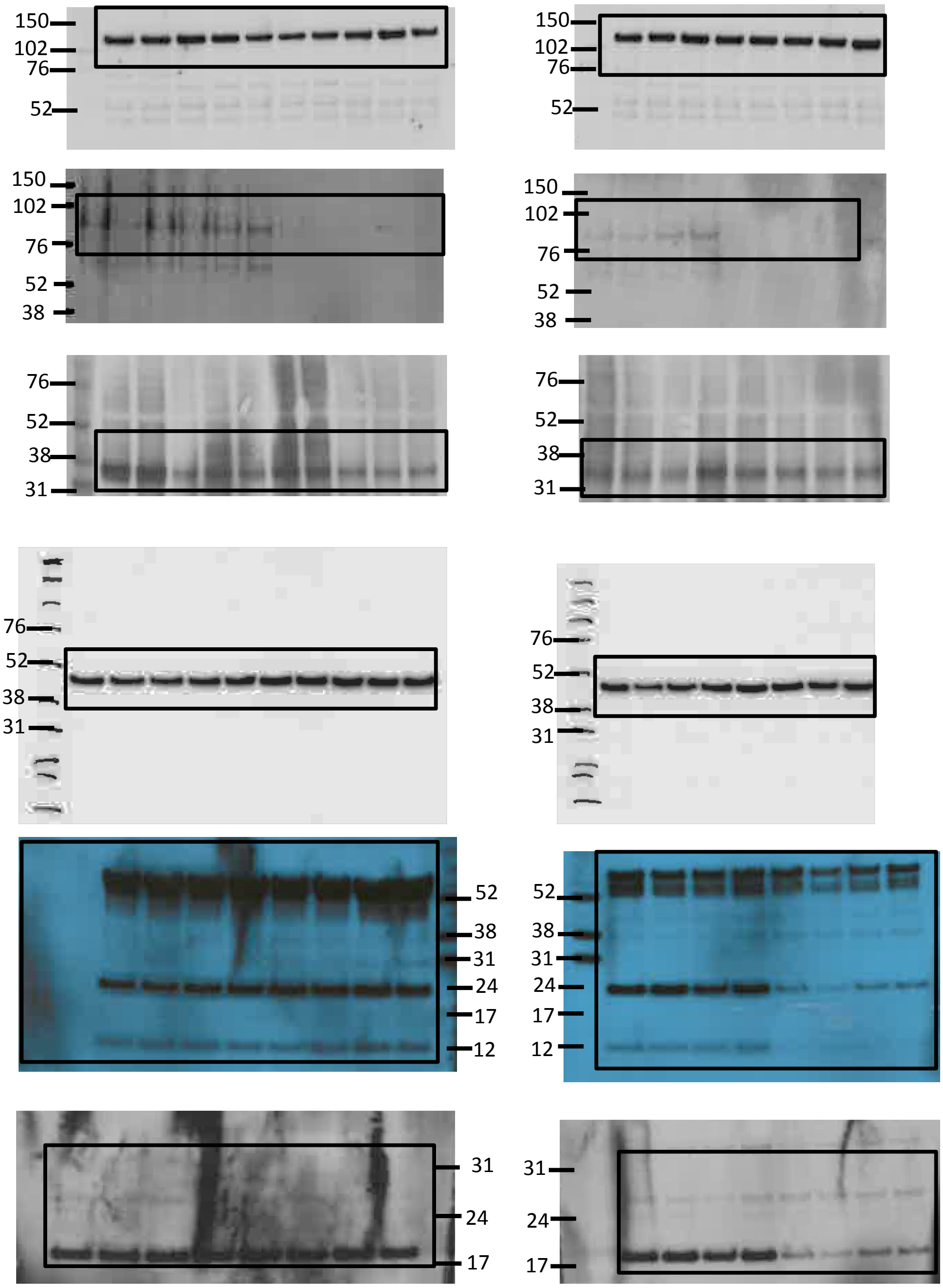

Fig. 2b (gel source data)

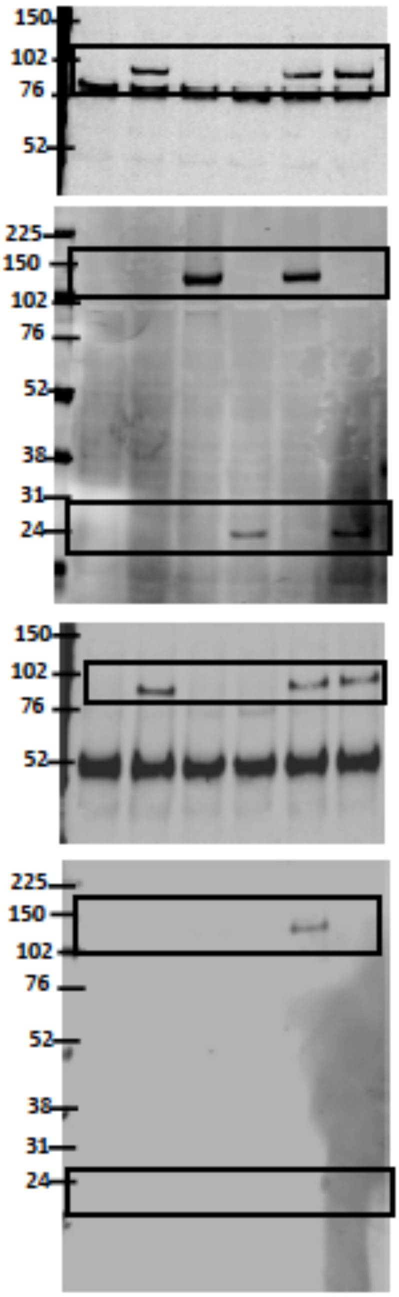

Fig. 3a (gel source data)

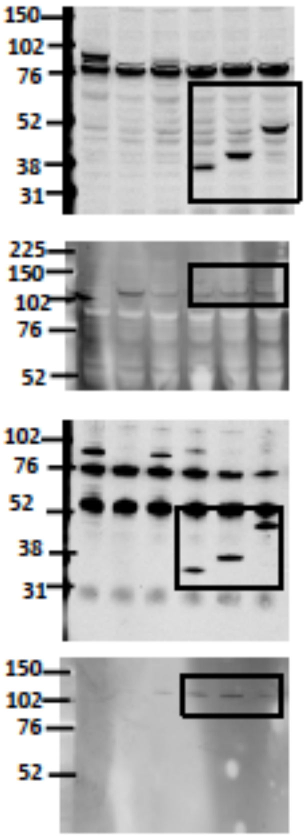

Fig. 3e (gel source data)

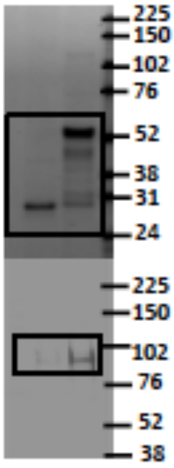

Fig. 3b (gel source data)

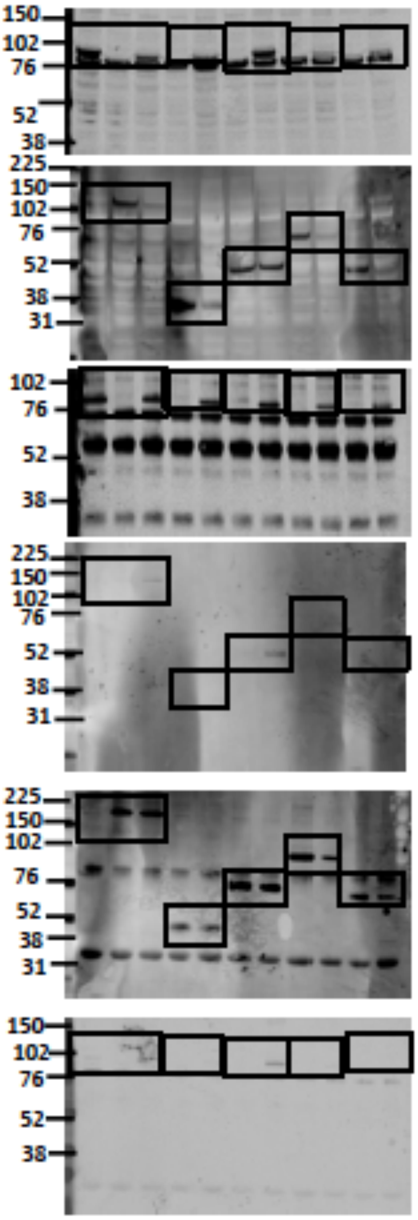

Fig. 3d (gel source data)

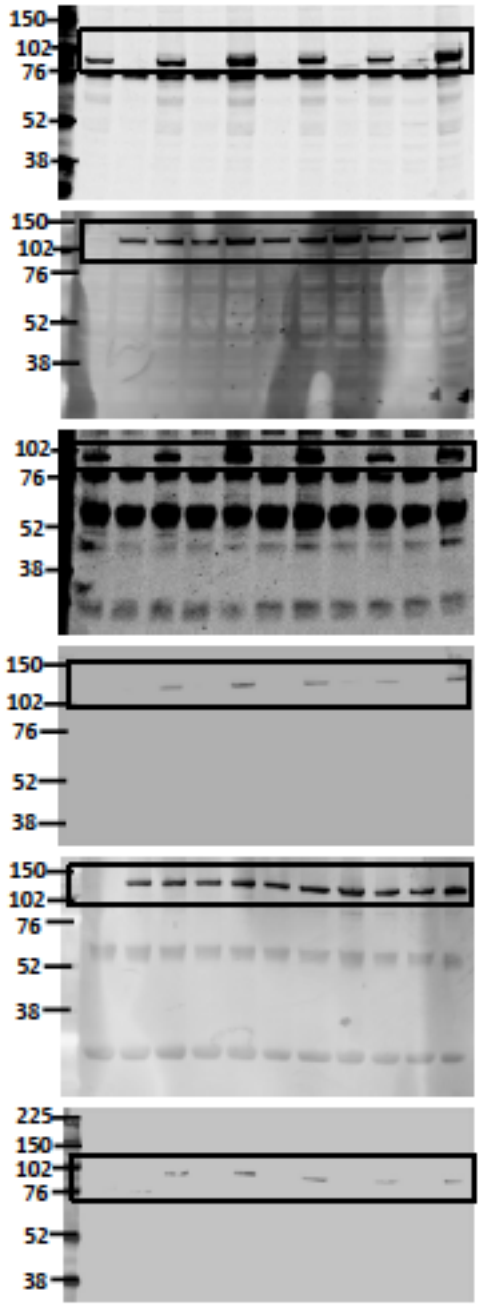

Supplementary Figure 17. Gel source data. The original western blot examples of Figure 1, 2 and 3 were displayed with molecular weight marker positions labeled on each blot.

Supplementary Movie 1. NLRP3 is moving along microtubule. PMA-differentiated THP-1 cells stably expressing Cherry-NLRP3 (red) were stained with TubulinTracker green to label microtubules (green), and Mitotracker Deep Red FM to label mitochondria (magenta). Arrow indicates a NLRP3 particle, and arrowhead indicates a mitochondrion. Cells were stimulated with nigericin (3 μM). Video corresponds to 105.27 seconds; width of the movie is 23.3 μm.

Supplementary Movie 2. Movement of NLRP3 in MARK4 knock-down cells. PMA-differentiated THP-1 cells stably expressing Cherry-NLRP3 and shRNA (scrambled control or MARK4) were stimulated with nigericin (3 μM). Video corresponds to 291 seconds; width of each movie is 25.5 μm.

Supplementary Movie 3. Knock-down of MARK4 affects translocation of NLRP3 on mitochondria. PMA-differentiated THP-1 cells stably expressing Cherry-NLRP3 (red) and shRNA (scrambled control or MARK4) were stimulated with nigericin (3 μM for 2 hrs), and then stained with mitotracker dye to label mitochondrion (green). Z section pictures were stacked to reconstitute 3D view.

Supplementary Movie 4. MARK4 and NLRP3 are moving together to MTOC. PMA-differentiated THP-1 cells stably expressing Cherry-NLRP3 (red) and GFP-MARK4 (green) were stimulated with nigericin (10 μM). Arrowhead indicates MTOC where MARK4 is accumulated. Video corresponds to 198.366 seconds; width of the movie is 30.3 μm.

Supplementary Movie 5. NLRP3 is shuttling towards MTOC. PMA-differentiated

THP-1 cells stably expressing Cherry-NLRP3 (red) were stained with Tubulin Tracker green to label microtubules (green) before or after nigericin stimulation (10 μM for 2 hrs). Z section pictures were stacked to reconstitute 3D view.

Supplementary Movie 6. Insufficient MARK4 causes dilated ring structure of NLRP3. HEK293T cells were co-overexpressed with GFP-MARK4, Cherry-NLRP3 and ASC-Flag (indicated as MARK4 GFP o.e.); or co-overexpressed with MARK4 shRNA (shown by green GFP), Cherry-NLRP3 and ASC-Flag (indicated as MARK4 shRNA). One day after expression, cells were subject to imaging. Z section pictures were stacked to reconstitute 3D view.
