# Supplementary material for: MARK4 regulates NLRP3 positioning and inflammasome activation through a microtubule-dependent mechanism
Source: Nat Commun. 2017 Jun 28;8:15986. doi: 10.1038/ncomms15986 (PMC5493753; doi:10.1038/ncomms15986)
Supplement: Peer Review File [file ncomms15986-s8.pdf]

Editorial Note: this manuscript has been previously reviewed at another journal that is not operating a transparent peer review scheme. This document only contains reviewer comments and rebuttal letters for versions considered at Nature Communications.

REVIEWERS' COMMENTS:

Reviewer #3 (Remarks to the Author):

The authors have adequately addressed my concerns and the manuscript has improved considerably.
This reviewer recommends it for publication.
